# The long noncoding RNA AC093895.1 promotes ovarian cancer formation and metastasis through a positive feedback network dependent on the transcription factor SOX4

**DOI:** 10.1038/s41419-026-08429-2

**Published:** 2026-02-03

**Authors:** Bin Huang, Honglin An, Yiman Qiu, Zhuona Ni, Liming Chen, Jiahui Lin, Shihan Lin, Han Wu, Hanqi Zhu, Yueting Fan, Shu Jiang, Yixin Chen, Wenqi Yu, Jiumao Lin

**Affiliations:** 1 The Affiliated People’s Hospital of Fujian University of Traditional Chinese Medicine, Fuzhou, China; 2 Academy of Integrative Medicine, Fujian Key Laboratory of Integrative Medicine on Geriatrics, Key laboratory of Integrative Medicine of Fujian Province University, Fujian University of Traditional Chinese Medicine, Fuzhou, China; 3 State Key Laboratory of Molecular Vaccinology and Molecular Diagnostics, National Institute of Diagnostics and Vaccine Development in Infectious Diseases, School of Life Sciences, School of Public Health, Xiamen University, Xiamen, China; 4 School of Medicine, Wuhan University of Science and Technology, Wuhan, China

**Keywords:** Cancer, Immunology

## Abstract

Recurrence and metastasis are the main causes of death in ovarian cancer (OC). Long non-coding RNAs (lncRNAs) are considered as good prognostic models and potential therapeutic targets for cancer patients because of their easy detection and strong correlation. Our study identifies an OC-associated lncRNA with tumor progression and therapeutic implications. It’s found that lncRNA AC093895.1 is highly expressed in OC tissues and correlated with poor prognosis. AC093895.1 has a potentiating effect during the progression and metastasis of ovarian cancer. The effects of AC093895.1 on ovarian cancer cells are miR-1253 dependent. Results showed that by interacting with tumor-suppressive gene miR-1253 as competing endogenous RNA (ceRNAs), AC093895.1 significantly upregulated the downstream gene SOX4 of AC093895.1/ miR-1253 axis, leading to tumor metastasis. In addition, chromatin immunoprecipitation (ChIP) results further confirmed that SOX4 could bind to the AC093895.1 promoter, forming a positive feedback loop SOX4/AC093895.1/miR-1253/SOX4. Therapeutic strategy to break the loop through AC093895.1 knockdown exhibited attenuated OC growth and metastasis in vivo both in SK-OV-3 subcutaneous model and pulmonary metastatic model. Our study unveiled the potentiating effects of SOX4/AC093895.1/miR-1253/SOX4 on ovarian cancer cell survival, migration, and invasion. AC093895.1 may be a promising patient prognostic biomarker and therapeutic candidate.

**Figure:**
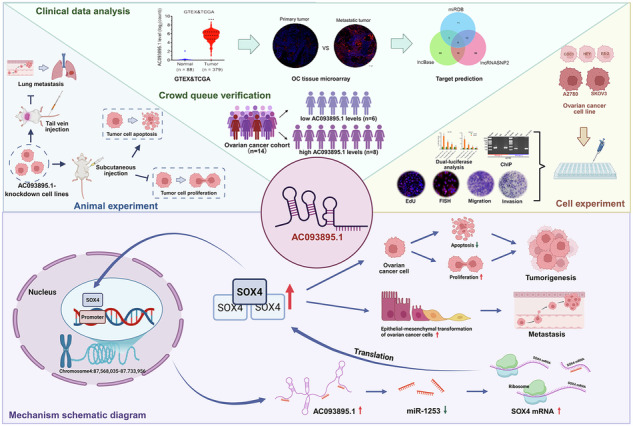
Created with BioRender.com.

Created with BioRender.com.

## Introduction

Ovarian cancer (OC) is a malignant tumor that ranks fifth in terms of cancer-related mortality among women, with over 20,000 new cases and at least 10,000 deaths each year [1]. Because the ovaries are located deep within the pelvis, early-stage OC often causes overlooked symptoms, hindering timely detection [2]. As a result, most patients with OC are diagnosed at the advanced stage, and 80% of them have already developed metastases, contributing to the high mortality rates [3]. Although clinical biomarkers such as CA125, HE4, and CD44 are used in practice, they still present certain limitations in evaluating treatment response and monitoring recurrence [4]. Moreover, lacking accurate prognostic prediction restricts the prompt detection of tumor progression, failing to provide guidance to the occurrence of OC metastasis and recurrence. Therefore, the identification of specific and accurate biomarkers for early-stage OC is crucial to improve clinical diagnosis and inform therapeutic strategies for mitigating malignancy.

Long noncoding RNAs (lncRNAs) are transcripts exceeding 200 nucleotides in length that lack protein-coding functions [5]. In recent years, lncRNAs have been found to play crucial roles in several diseases and serve as classifiers for personalized cancer therapy [6, 7]. Gene expression can be regulated by lncRNAs through diverse mechanisms [8], including interaction with transcription factors and chromatin modifiers to modulate transcriptional events [9], function as molecular sponges through a “competing endogenous RNA (ceRNA)” mechanism to target microRNAs (miRNAs) for mRNA stability regulation [10–12], etc. Moreover, anomalies in the expression and regulation of lncRNAs are reported to be correlated with tumor recurrence and metastasis. And owing to the high detection sensitivity, lncRNAs are increasingly regarded as promising prognostic biomarkers and potential targets for cancer treatment [13–16].

As key components of the ceRNA network, microRNAs (miRNAs) represent a class of endogenous non-coding RNA molecules approximately 22 nucleotides in length that regulate gene expression at the post-transcriptional level. They play extensive roles in various biological processes, including proliferation, differentiation, metabolism, and apoptosis of tumor cells, exerting crucial regulatory functions in tumorigenesis and progression [17, 18]. Among them, miR-1253 functions as a tumor suppressor in multiple cancers [19–22]. However, its biological role in ovarian cancer remains largely unexplored. SOX4 is a pluripotent transcription factor that drives malignant progression in multiple cancers by promoting cell proliferation, survival, invasion, and metastasis. Its elevated expression is closely correlated with advanced clinical stage, lymph node metastasis, and unfavorable prognosis [23].

The lncRNA AC093895.1 is a newly characterized intergenic noncoding RNA located on chromosome 4 which may be involved in tumor development. However, its precise role in tumor biology remains unknown [24, 25]. This study demonstrates for the first time that AC093895.1 plays a pivotal role in OC recurrence and metastasis, and discovers the novel SOX4/AC093895.1/miR-1253/SOX4 loop. It’s found that AC093895.1 interacts with tumor-suppressive gene miR-1253 as ceRNAs, and inhibition of miR-1253 further upregulates the downstream gene SOX4 of AC093895.1/miR-1253 axis, which promotes the proliferation and metastasis of A2780 and SK-OV-3 cells. Furthermore, SOX4 further binds to the AC093895.1 promoter, forming a positive feedback loop SOX4/AC093895.1/miR-1253/SOX4. Animal experiments exhibited significant anti-tumor and anti-metastasis results in vivo after the feedback loop was disrupted by AC093895.1 knockdown.

This study underscores the pivotal role of lncRNA AC093895.1 in OC, and illuminates the mechanisms driving OC progression and metastasis, which firstly unveils the positive feedback loop SOX4/AC093895.1/miR-1253/SOX4 for potential intervention. Furthermore, our findings provide novel clinical prognostic biomarker panels with high detection accuracy and sensitivity. They also shed light on new therapeutic strategies to reduce OC recurrence and metastasis.

## Results

### High AC093895.1 levels are linked to ovarian cancer metastasis and a poor prognosis

**Fig. 1:**
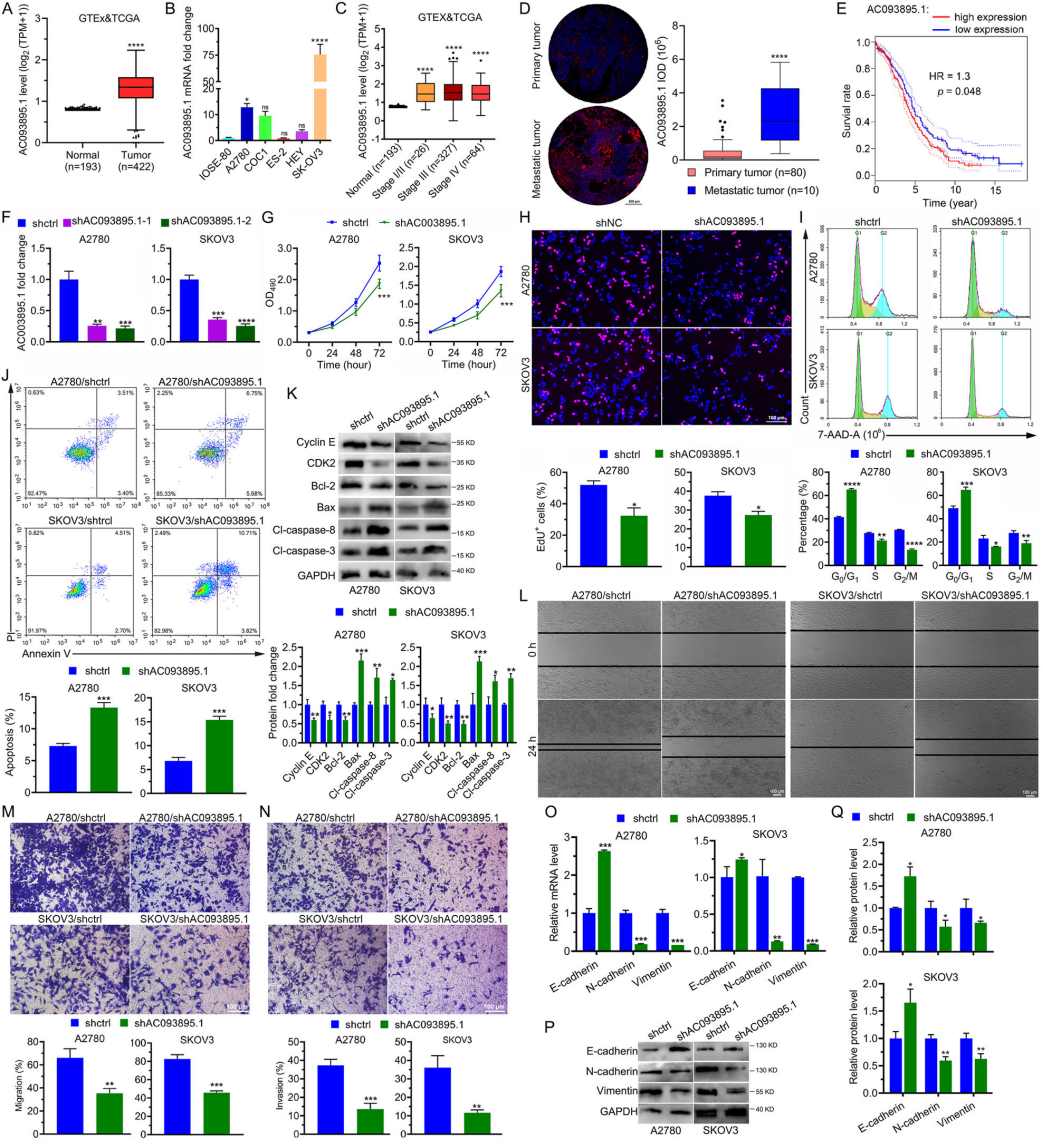
High expression of AC093895.1 in ovarian cancer inhibits apoptosis and enhances cell viability, migration, and invasion. **A** Differential expression of the lncRNA AC093895.1 between normal ovarian (*n* = 193) and ovarian cancer (*n* = 422) tissues on the basis of transcriptomic data from the GTEx and TCGA databases. **B** RT-qPCR analysis of AC093895.1 in healthy ovarian cells, IOSE-80, and the ovarian carcinoma cell lines A2780, COC1, ES-2, HEY, and SK-OV-3. Statistical analysis was performed relative to AC093895.1 levels in IOSE-80 cells. **C** Differential expression analysis of AC093895.1 in patients with ovarian cancer at different clinical stages. Analysis was performed on the basis of transcriptomic data from the GTEx and TCGA databases. Statistical analysis was performed relative to AC093895.1 levels in healthy individuals. **D** Tissue microarray analysis revealed differential expression of AC093895.1 in primary and metastatic tumors. **E** The survival rates of patients with ovarian cancer were dependent on AC093895.1 expression, as shown by the Kaplan–Meier results. **F** RT-qPCR results of AC093895.1 levels in A2780 and SK-OV-3 cells with AC093895.1 knockdown via two shRNAs. Statistical analysis was performed relative to the AC093895.1 level in the control cells (shCtrl) (*n* = 3/group). **G** MTT results showed mitigated cell proliferation upon AC093895.1 knockdown (*n* = 3/group). **H** Typical images of EdU+ cells depleted of AC093895.1 (*n* = 3/group). **I** Typical images of the cell cycle phases and their quantification via flow cytometry after AC093895.1 knockdown in A2780 and SK-OV-3 cells (*n* = 3/group). **J** Cell apoptosis and quantification via flow cytometry after AC093895.1 was knocked down in ovarian cancer cells (*n* = 3/group). **K** Western blotting of apoptosis- and cell cycle-related proteins in ovarian cancer cells upon AC093895.1 knockdown (*n* = 3/group). **L** Typical images of wound healing assays of ovarian cancer cells before and after AC093895.1 knockdown (*n* = 3/group). Typical images and quantification of Transwell results for detecting the migration (**M**) and invasion (**N**) of cancer cells depleted of AC093895.1 (*n* = 3/group). RT-qPCR (**O**) and WB (**P**, **Q**) of EMT markers in ovarian cancer cells upon AC093895.1 knockdown (*n* = 3/group). The t-test was applied to check the statistical difference: *P < 0.05, **P < 0.01, ***P < 0.001, ****P < 0.0001.

Transcriptomic data from the Genotype-Tissue Expression (GTEx) and Cancer Genome Atlas (TCGA) databases were analyzed to evaluate AC093895.1 levels in tissues from patients with OC and healthy individuals. As expected, AC093895.1 expression was markedly increased in tumors (Fig. 1A). Compared with that in the normal ovarian epithelial cell line IOSE-80, the expression of AC093895.1 was markedly higher in the OC cell lines A2780, COC1, HEY, and SK-OV-3 (Fig. 1B). Although the levels of AC093895.1 were markedly elevated at different cancer stages, no significant difference was observed among the stages (Fig. 1C). Notably, the tissue microarray results indicated that AC093895.1 levels were further increased in metastatic tumors (Fig. 1D). The Kaplan–Meier survival curves demonstrated that patients with higher AC093895.1 levels had poor outcomes compared to those with lower AC093895.1 levels (Fig. 1E). These results indicate that AC093895.1 is highly expressed in metastatic OC, and patients with high AC093895.1 levels have poor outcomes, demonstrating that AC093895.1 may serve as a prognostic indicator for OC patients.

### Knockdown of AC093895.1 in ovarian cancer cells reduces cell viability, migration and invasion while promoting cell apoptosis

Considering the upregulation of AC093895.1 in OC and its increased expression in metastases, we hypothesized that its downregulation would mitigate the progression of this disease. To test this, AC093895.1 was depleted in A2780 and SK-OV-3 cells using two different shRNAs (Fig. 1F). MTT and EdU assays revealed that AC093895.1 knockdown significantly reduced cell proliferation (Fig. 1G, H). Furthermore, cell cycle analysis indicated that AC093895.1 depletion induced a G0/G1 phase arrest (Fig. 1I). We subsequently assessed whether AC093895.1 affected cell apoptosis and found that the ratio of apoptotic cells increased after AC093895.1 knockdown (Fig. 1J). Western blot analysis revealed increased expression of Bax, cleaved caspase-8, and cleaved caspase-3, and decreased levels of Cyclin E1, CDK2, and Bcl-2 (Fig. 1K) after AC093895.1 knockdown. Collectively, these findings demonstrate that AC093895.1 knockdown impairs cell viability, arrests cell cycle at G0/G1 phase, and promotes cell apoptosis of OC cells.

Furthermore, the invasive and migratory abilities of A2780 and SK-OV-3 cells were attenuated following AC093895.1 depletion (Fig. 1L–N). Given that EMT promotes cell invasion and migration, the expression of EMT markers was assessed by Western blotting. Results suggested that AC093895.1 knockdown upregulated E-cadherin levels and downregulated mesenchymal markers, such as vimentin and N-cadherin (Fig. 1O–Q). These findings demonstrate that AC093895.1 knockdown decreases OC cell invasion and migration.

### AC093895.1 interacts with miR-1253

**Fig. 2:**
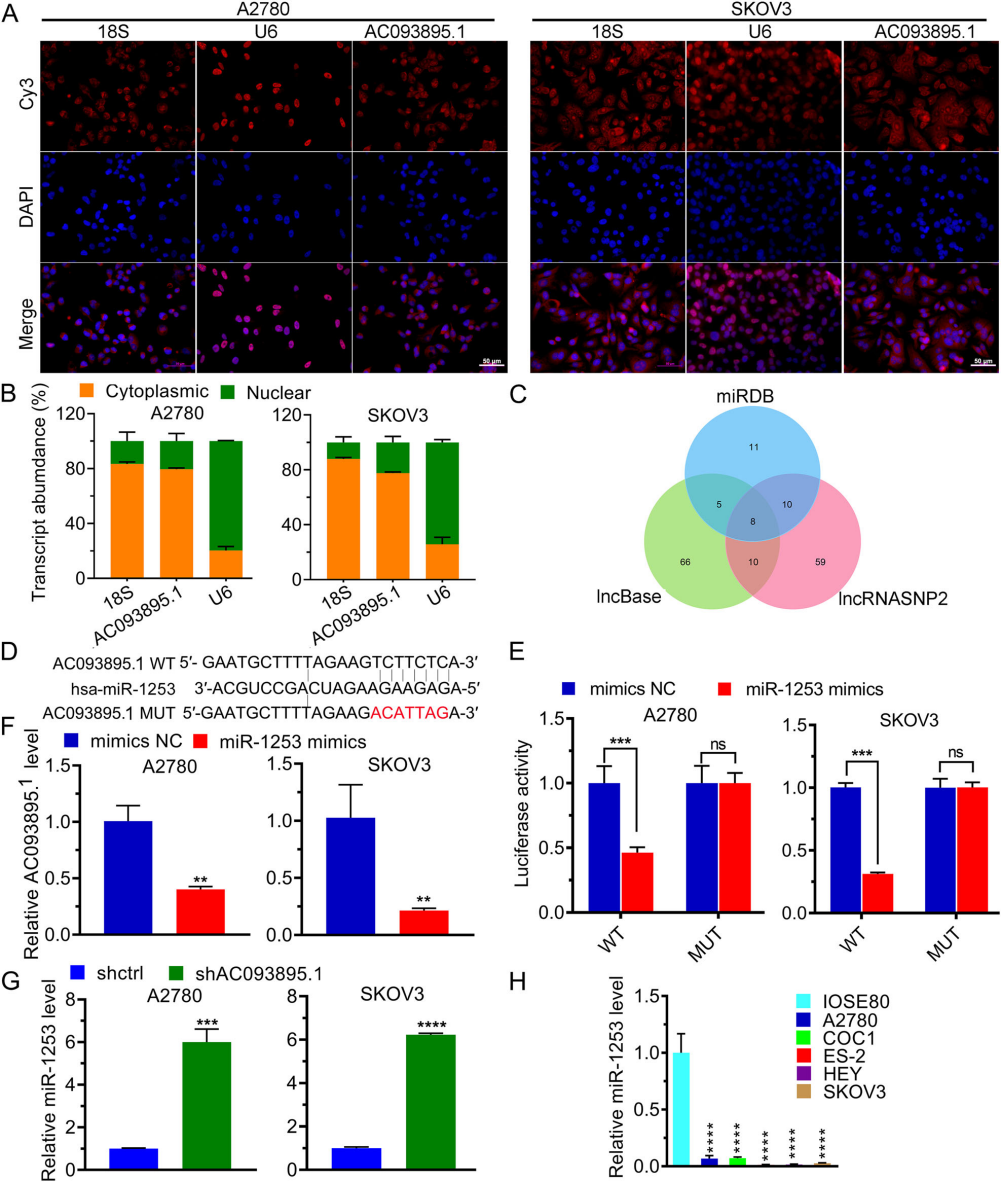
miR-1253 is a miRNA target of AC093895.1. **A** Representative images of fluorescence in situ hybridization using probes against the indicated RNAs (*n* = 3/group). **B** RT-qPCR quantification of the subcellular fractionation results (*n* = 3/group). **C** Venn diagram suggest the intersections among these 3 databases used for predicting the downstream miRNA targets of AC093895.1. **D** DNA sequences of the putative binding site between AC093895.1 and the wild-type (WT) or mutant (MUT) miR-1253. **E** Dual-luciferase analysis of the relative luciferase activities of the indicated reporters in mock- or miR-1253-transfected ovarian cancer cells (*n* = 3/group). **F** After the overexpression of miR-1253, the AC093895.1 level was detected via RT-qPCR (*n* = 3/group). **G** After AC093895.1 knockdown, the level of miR-1253 was determined by RT-qPCR (*n* = 3/group). **H** RT-qPCR results of miR-1253 in IOSE-80 and A2780 cells and COC1, ES-2, HEY, and SK-OV-3 cells. Statistical analysis was performed relative to miR-1253 levels in IOSE-80 cells (*n* = 3/group). A t-test was used to determine significant differences: **P < 0.01, ***P < 0.001, ****P < 0.0001.

The subcellular localization of AC093895.1 was analyzed via FISH and was found to be primarily localized in the cytoplasm (Fig. 2A). Subcellular fractionation followed by RT-qPCR further confirmed its subcellular localization (Fig. 2B), indicating that AC093895.1 may bind to miRNAs. After mining the miRDB, lncBase, and lncRNASNP2 databases, eight miRNAs were identified as bona fide interactors of AC093895.1 (Fig. 2C). Considering the tumor-suppressive functions of miR-1253 in breast, colon, and lung cancers [26–28], we evaluated its interaction with AC093895.1. The interaction between miR-1253 and AC093895.1 was confirmed by dual-luciferase reporter assay (Fig. 2D, E), and further supported by RNA pull-down assays showing specific enrichment of AC093895.1 with the wild-type miR-1253 probe (Fig. S1). Moreover, reduced AC093895.1 expression was observed in cells transfected with the miR-1253 mimic (Fig. 2F), whereas miR-1253 levels were elevated in the absence of AC093895.1 (Fig. 2G). In addition, the miR-1253 levels were markedly lower in A2780, COC1, ES-2, HEY, and SK-OV-3 cells compared to IOSE-80 cells (Fig. 2H). Collectively, these results imply that AC093895.1 and miR-1253 negatively regulate each other.

### Inhibition of miR-1253 alleviates the biological effects triggered by AC093895.1 knockdown

**Fig. 3:**
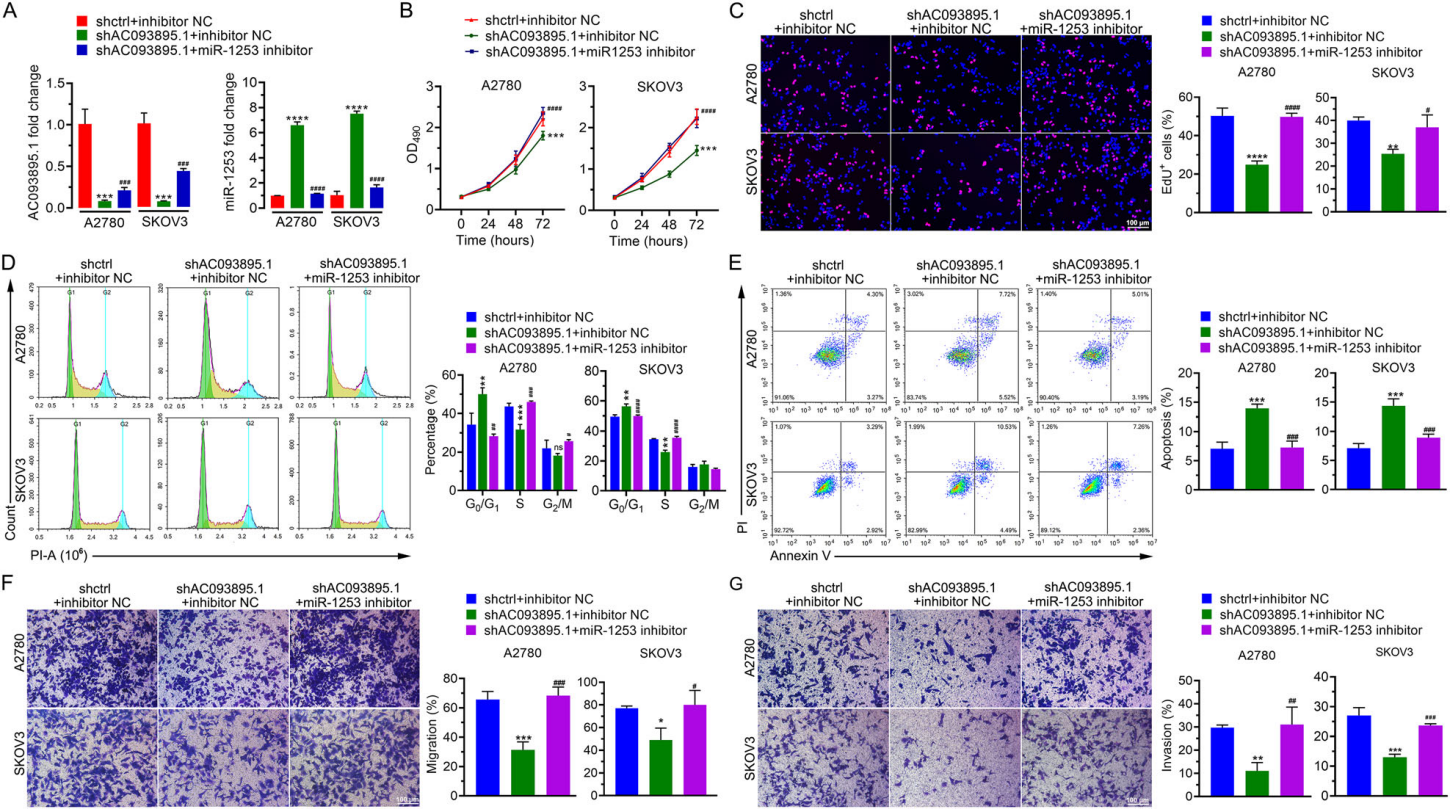
The effects of AC093895.1 on ovarian cancer cell survival, apoptosis, migration, and invasion are miR-1253 dependent. **A** The expression of AC093895.1 and miR-1253 in ovarian cancer cell lines after AC093895.1 knockdown and/or miR-1253 inhibition was analyzed via RT-qPCR (*n* = 3/group). **B** Cell proliferation after AC093895.1 knockdown or miR-1253 inhibition was detected via MTT assays (*n* = 3/group). **C** Typical images and quantification of EdU+ cells with AC093895.1 knockdown or miR-1253 inhibition (*n* = 3/group). **D** Representative images of the cell cycle and their quantification via flow cytometry in AC093895.1-knockdown or miR-1253-inhibited cells (*n* = 3/group). **E** Typical images of cell apoptosis and relevant quantification via flow cytometry in AC093895.1 knockdown or miR-1253 inhibition cancer cells (*n* = 3/group). Transwell results related to the migration (**F**) and invasion (**G**) of AC093895.1 knockdown or miR-1253 inhibition cancer cells (*n* = 3/group). The t-test and ANOVA were applied to check statistical difference: *P < 0.05, **P < 0.01, ***P < 0.001 in comparison with the shctrl + inhibitor NC group; # *P* < 0.05, ## *P* < 0.01, ### *P* < 0.001 in comparison with the shAC093895.1 + inhibitor NC group.

Cells with AC093895.1 depletion were treated with miR-1253 inhibitors, which significantly reversed the upregulation of miR-1253 induced by AC093895.1 depletion (Fig. 3A). Additionally, the miR-1253 inhibitor restored cell viability (Fig. 3B, C) and alleviated G0/G1 phase arrest caused by AC093895.1 knockdown (Fig. 3D). Furthermore, the miR-1253 inhibitor decreased apoptosis and improved the migration and invasion of AC093895.1-depleted OC cells (Fig. 3E–G). These results indicate that the effects of AC093895.1 on viability, migration, and invasion are miR-1253 dependent.

### SOX4 is a direct downstream gene of the AC093895.1/miR-1253 axis

**Fig. 4:**
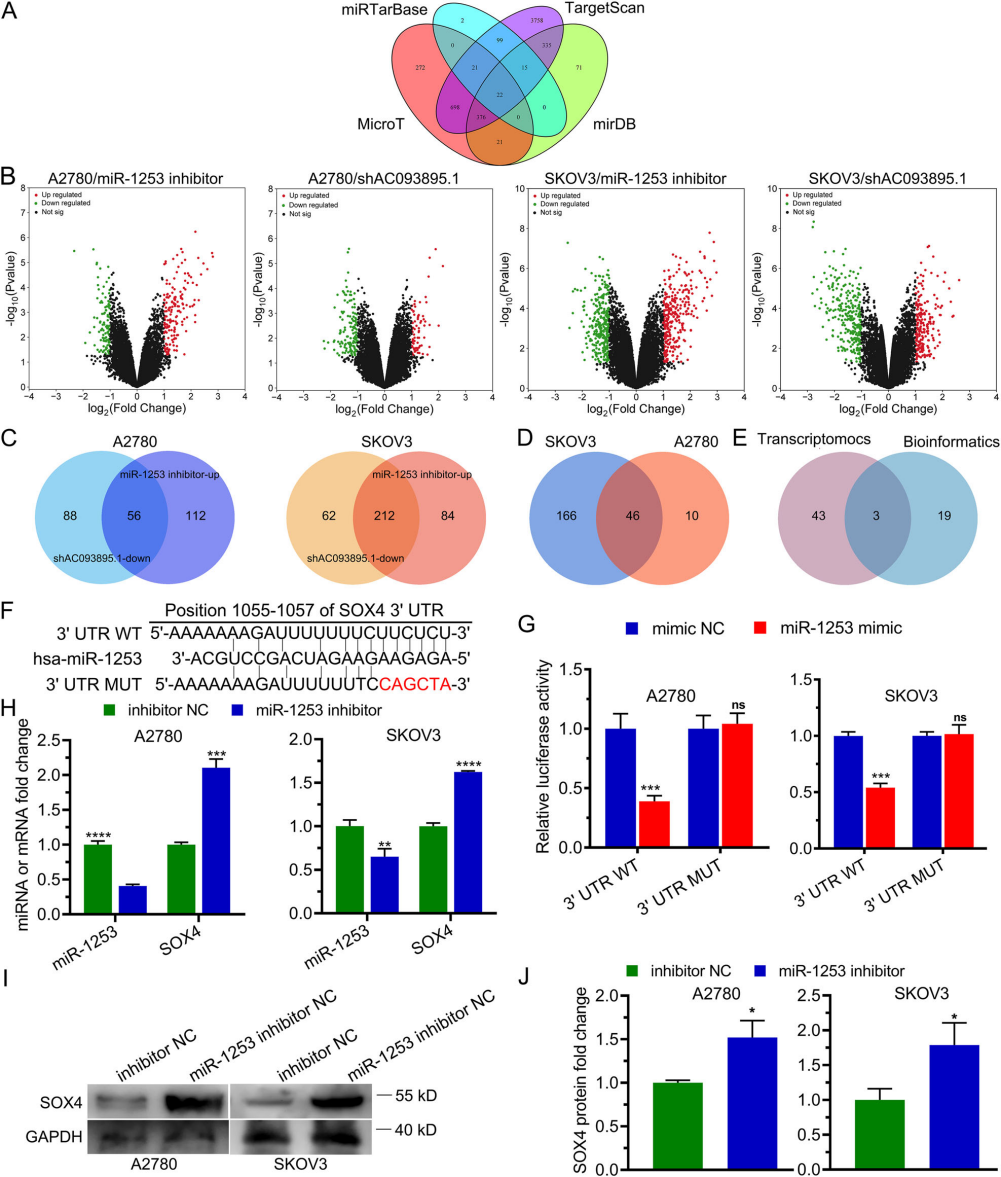
SOX4 is one of the downstream genes of both AC093895.1 and miR-1253. **A** Venn diagram showing the intersections among the 4 databases used to predict the downstream mRNA targets of miR-1253. **B** Volcano plots of DEGs in A2780 and SK-OV-3 cells with AC093895.1 knockdown or miR-1253 inhibition (*n* = 3/group). **C**, **D** Intersection analysis of DEGs in A2780 and SK-OV-3 cells with AC093895.1 knockdown or miR-1253 inhibition (*n* = 3/group). **E** Intersection analysis of the transcriptomics results and bioinformatic analysis results. **F** DNA sequences of the putative binding site between miR-1253 and the wild-type (WT) or mutant (MUT) 3′ UTR of SOX4. **G** Dual-luciferase reporter analysis of the relative luciferase activities of the indicated reporters in miR-1253-overexpressing ovarian cancer cells (*n* = 3/group). **H** RT-qPCR quantification of miR-1253 and SOX4 expression in ovarian cancer cells with miR-1253 inhibition (*n* = 3/group). **I**, **J** Western blotting analysis of SOX4 levels in ovarian cancer cells transfected with a miR-1253 inhibitor (*n* = 3/group). A t-test was used to assess significant differences: *P < 0.05, **P < 0.01, ***P < 0.001.

Using the miRTarBase, TargetScan, MicroT, and miRDB databases, we identified 22 potential downstream genes that may directly interact with miR-1253 (Fig. 4A). To further pinpoint key targets of both AC093895.1 and miR-1253, transcriptome sequencing was performed in A2780 and SK-OV-3 cells following AC093895.1 knockdown or miR-1253 inhibition, and differentially expressed genes (DEGs) were screened (Fig. 4B). Among the overlapping DEGs, SOX4, SEMA7A, and PPP1CC were identified as common downstream gene targets of AC093895.1 and miR-1253 (Fig. 4C–E).

Given that SOX4 is the only transcription factor among the identified genes [29] and that the TCGA and GTEx datasets revealed elevated SOX4 expression in OC tissues compared with normal ovarian tissues, we selected SOX4 for further experiments. The dual-luciferase reporter assay results revealed that miR-1253 bound specifically to wild-type SOX4 mRNA but not to its mutant form (Fig. 4F, G). Moreover, both SOX4 mRNA and protein levels were increased after miR-1253 inhibition (Fig. 4H–J). Notably, given the considerable basal expression of SOX4, we reasoned that the endogenous miR-1253 might be functionally saturated in regulating its target SOX4.

### AC093895.1 positively regulates SOX4 by targeting miR-1253, while SOX4 directly upregulates AC093895.1 expression

**Fig. 5:**
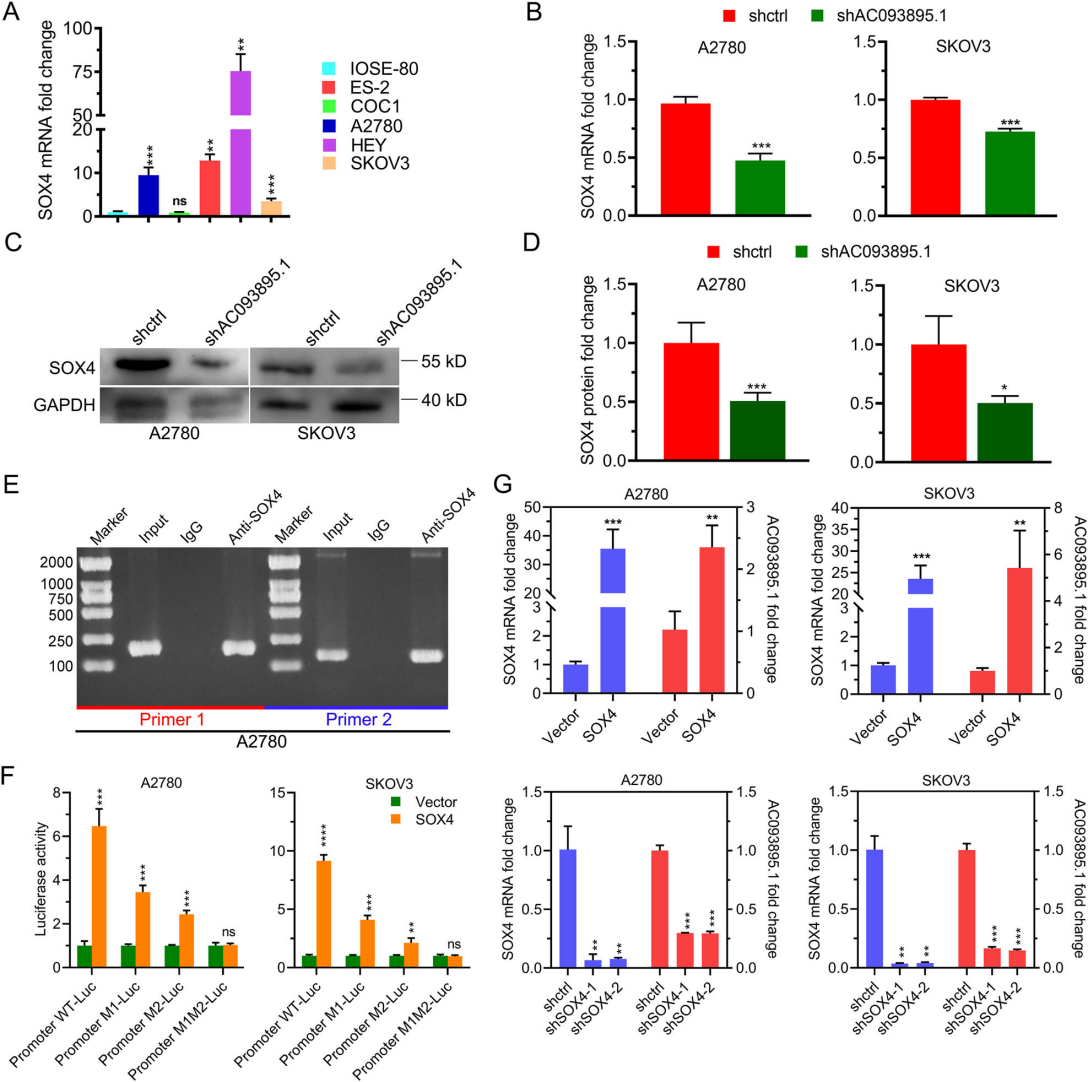
SOX4 is an indirect target of AC093895.1 and can also bind to the promoter sequence of AC093895.1. **A** RT-qPCR analysis of SOX4 in the IOSE-80 and A2780, COC1, ES-2, HEY, and SK-OV-3 cell lines. Statistical analysis was performed relative to the SOX4 level in IOSE-80 cells (*n* = 3/group). RT-qPCR quantification (**B**) and WB (**C**, **D**) of SOX4 in ovarian cancer cells after AC093895.1 knockdown (*n* = 3/group). **E** Agarose gel images showing the immunoprecipitated DNA fragments containing the AC093895.1 promoter immunoprecipitated with the anti-SOX4 antibody. The IgG antibody was used as the negative control. **F** Dual-luciferase analysis of the relative luciferase activities of the indicated reporters in mock- or SOX4-transfected ovarian cancer cells (*n* = 3/group). **G** The expression levels of SOX4 and AC093895.1 were detected via RT-qPCR in ovarian cancer cells with altered SOX4 expression (overexpressing or depleted) (*n* = 3/group). Student’s *t* test was used to evaluate significant differences: *P < 0.05, **P < 0.01, ***P < 0.001.

According to the RT-qPCR results, SOX4 expression levels were higher in A2780, ES-2, HEY, and SK-OV-3 cells than in IOSE-80 cells. Therefore, A2780 and SK-OV-3 cells were selected for subsequent experiments (Fig. 5A). Given that miR-1253 is negatively regulated by AC093895.1 (Figs. 2–4), we next investigated whether AC093895.1 indirectly regulated the expression of SOX4. Results found that SOX4 levels were significantly reduced in AC093895.1-depleted OC cells relative to controls (Fig. 5B–D). These findings suggest that AC093895.1 positively regulates SOX4 expression by suppressing miR-1253 expression.

Since SOX4 is a transcription factor [23], we performed chromatin immunoprecipitation (ChIP) assay [30] to determine whether it directly binds to the AC093895.1 promoter. Compared with the IgG antibody negative control, the anti-SOX4 antibody precipitated DNA fragments containing the AC093895.1 promoter (Fig. 5E). Dual-luciferase reporter assays further confirmed that the promoter region contained two SOX4 binding sites (Fig. 5F). Additionally, the RT-qPCR results demonstrated that SOX4 overexpression increased AC093895.1 levels in A2780 and SK-OV-3 cells. Conversely, AC093895.1 levels decreased with decreasing SOX4 expression (Fig. 5G). Collectively, these findings suggest that SOX4 directly upregulates AC093895.1 transcription.

### A reduction in AC093895.1 expression alleviates the proliferation and metastasis of cancer cells caused by SOX4 overexpression

**Fig. 6:**
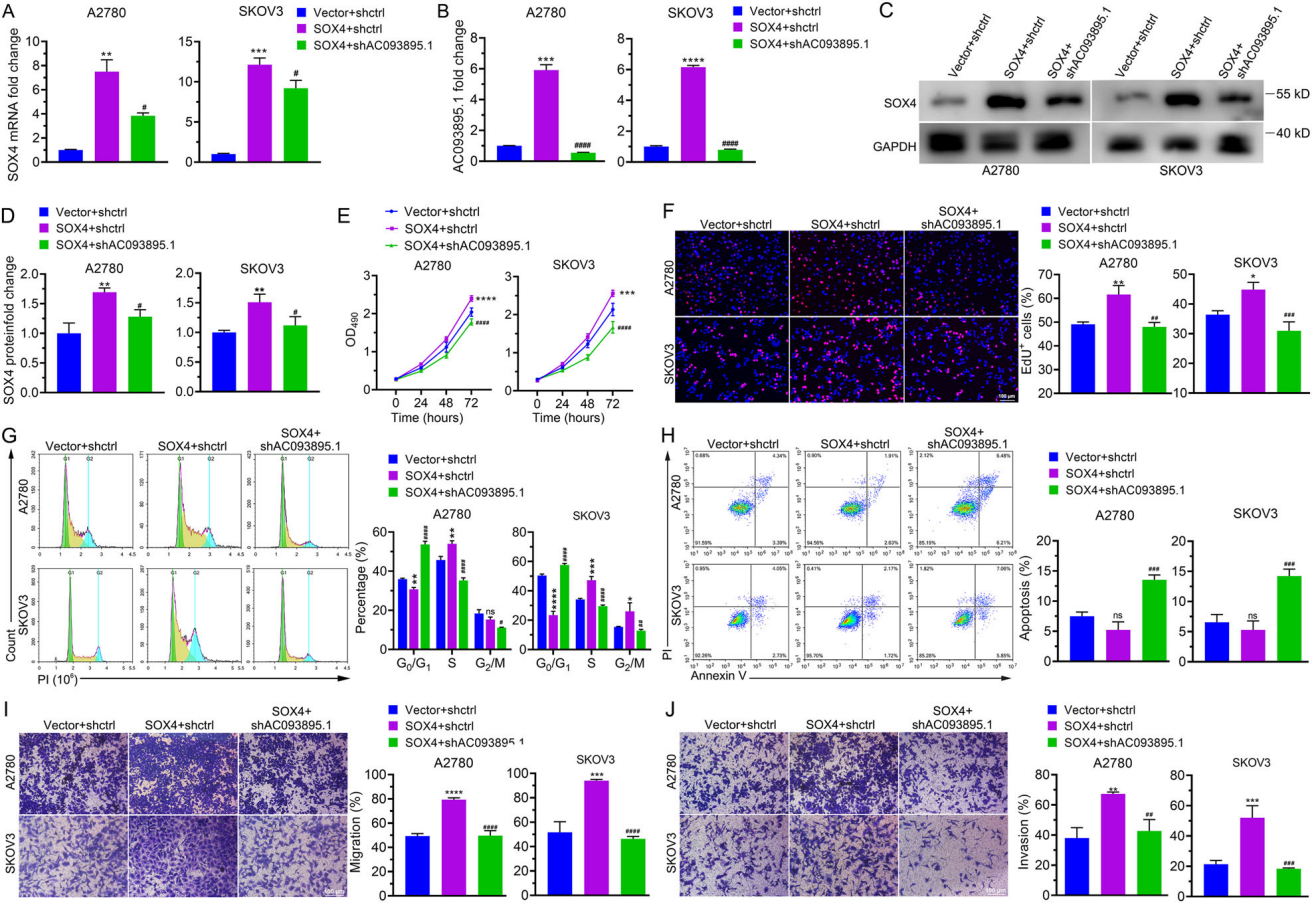
AC093895.1 is required for SOX4-mediated inhibition of ovarian cancer cell apoptosis, increase in cell viability and cell migration and invasion. RT-qPCR analysis of SOX4 (**A**) and AC093895.1 (**B**) levels in SOX4-overexpressing or AC093895.1-knockdown ovarian cancer cell lines (*n* = 3/group). **C**, **D** Quantification of SOX4 via western blotting in SOX4-overexpressing or AC093895.1-knockdown ovarian cancer cells (*n* = 3/group). **E** Cell proliferation was detected via MTT assays upon SOX4 overexpression or AC093895.1 knockdown (*n* = 3/group). **F** Typical images and quantification of EdU+ cells overexpressing SOX4 or lacking AC093895.1 (*n* = 3/group). **G** Representative images (top) of the cell cycle phases and their quantification (bottom) via flow cytometry in SOX4-overexpressing or AC093895.1-knockdown cells (*n* = 3/group). **H** Typical images and quantification of cell apoptosis via flow cytometry in SOX4-overexpressing or AC093895.1-knockdown ovarian cancer cells (*n* = 3/group). Typical images and quantification of the migration (**I**) and invasion (**J**) of cells overexpressing SOX4 or lacking AC093895.1 (*n* = 3/group). A t-test and ANOVA were employed to evaluate significant differences: *P < 0.05, **P < 0.01, ***P < 0.001, compared with the Vector + shctrl group; # *P* < 0.05, ## *P* < 0.01, ### *P* < 0.001, compared with the SOX4 + shctrl group.

To determine whether AC093895.1 is a downstream target of SOX4, we examined the effects of AC093895.1 knockdown in the context of SOX4 overexpression. Silencing AC093895.1 downregulated ectopically expressed SOX4 mRNA and attenuated SOX4-induced AC093895.1 upregulation in OC cell lines (Fig. 6A–D). In the absence of AC093895.1, the promoting effect of SOX4 on cell proliferation and cell cycle progression of OC cells was also affected (Fig. 6E–G). Furthermore, AC093895.1 knockdown mitigated the decrease in apoptosis and increase in cell migration and invasion caused by increased SOX4 levels (Fig. 6H–J). Collectively, these results indicate that the knockdown of AC093895.1 alleviated the increased proliferation and metastatic ability of cancer cells caused by SOX4 overexpression.

### In situ multicolor IF confirmed the interrelationships in the SOX4/AC093895.1/miR-1253/SOX4 loop

**Fig. 7:**
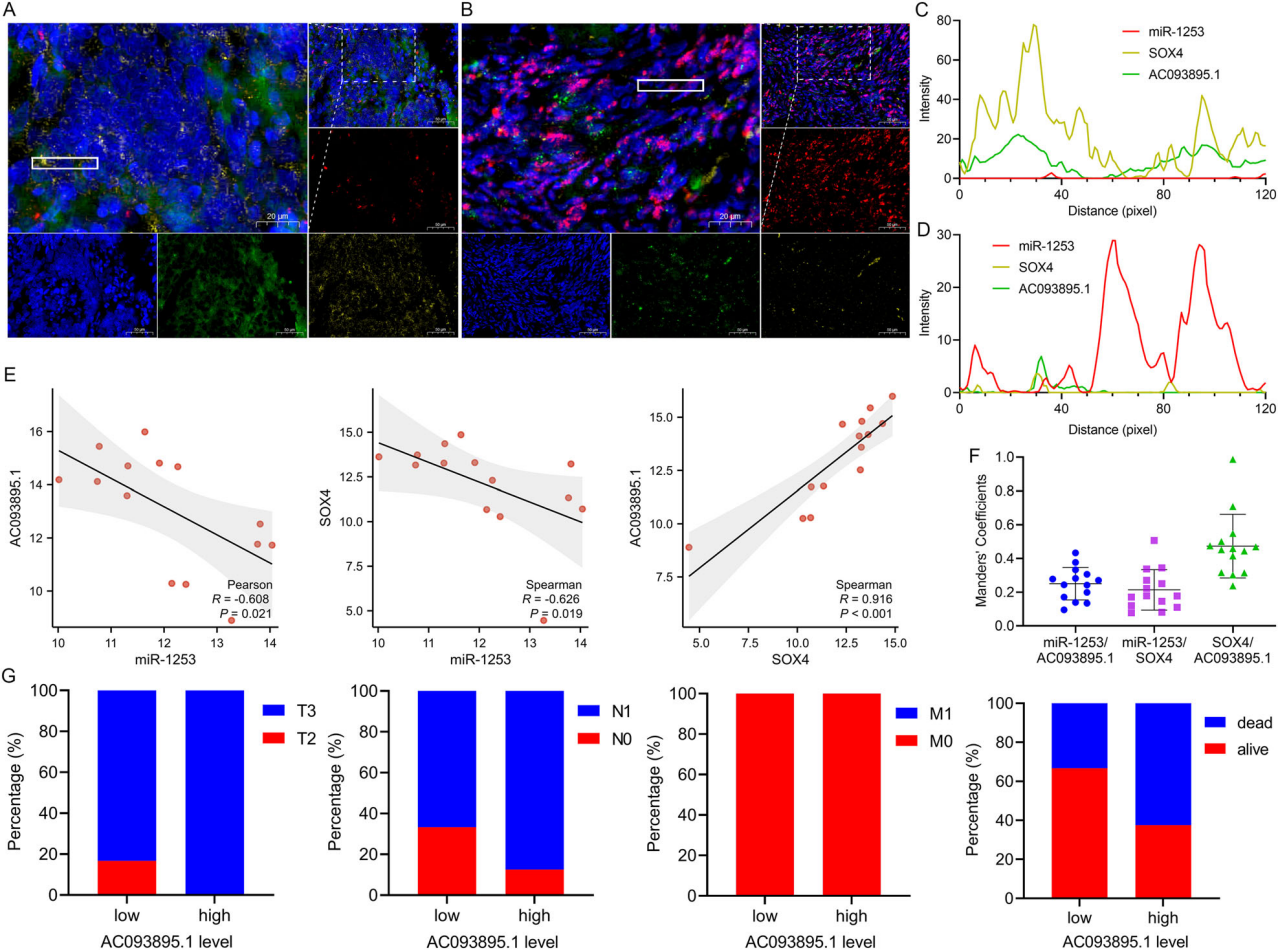
The SOX4/AC093895.1/miR-1253/SOX4 loop is found in ovarian cancer, linking high AC093895.1 levels to more aggressive TNM stages. **A**, **B** Representative images of AC093895.1 (green), miR-1253 (red) and SOX4 (yellow) staining in OC tissues from our cohort (*n* = 14). Scale bars, 20 μm and 50 μm. **C**, **D** Colocalization analysis of positive signals. **E**, **F** Correlations and Mander’s coefficients among AC093895.1, miR-1253 and SOX4 in OC tissues from the cohort. Association statistical analyses were conducted, employing Pearson’s method for normally distributed data, whereas Spearman’s approach was utilized for nonnormally distributed data. *P* < 0.05 was considered significant. **G** Quantification of staining in tissues from patients with OC with high AC093895.1 levels (*n* = 8) and low AC093895.1 levels (*n* = 6) in relation to the TNM stage.

To investigate the clinical and pathological relevance of the SOX4/AC093895.1/miR-1253/SOX4 loop in OC progression, we analyzed a cohort of 14 OC patients. Expression levels of AC093895.1, miR-1253, and SOX4 in OC tissues were assessed via in situ multicolor immunofluorescence, which was characterized by high AC093895.1 expression, elevated expression of SOX4 and decreased expression of miR-1253. Colocalization analysis revealed that the expression pattern of AC093895.1 aligned with that of SOX4 but was not related to that of miR-1253 (Fig. 7A, C). Conversely, tissues with elevated miR-1253 expression displayed reduced levels of both AC093895.1 and SOX4, and the increase in miR-1253 was not associated with changes in AC093895.1 or SOX4 (Fig. 7B, D). Correlation and Mander’s coefficient analyses indicated that AC093895.1 and SOX4 levels were inversely related to miR-1253, while AC093895.1 was directly correlated with SOX4 (Fig. 7E, F). These results confirm the clinical presence of the SOX4/AC093895.1/miR-1253/SOX4 feedback loop in OC.

In addition, of the 14 clinical samples, 8 were classified as high expression AC093895.1 and 6 as low expression. All patients with elevated AC093895.1 in the primary tumor (T category) were in the T3 stage, surpassing the percentage in the low-expression group. A significant proportion (87.5%) of these patients were in the N1 stage of regional lymph nodes (N category), and 62.5% of patients died from the disease, both rates exceeding those of patients with lower AC093895.1 expression (Fig. 7G). Moreover, patients with high expression of AC093895.1 had significantly higher rates of organ and lymph node metastasis than those with low expression (Table S2–S3).

### Inhibition of AC093895.1 blocked the SOX4/AC093895.1/miR-1253/SOX4 loop, tumor growth and lung metastasis

**Fig. 8:**
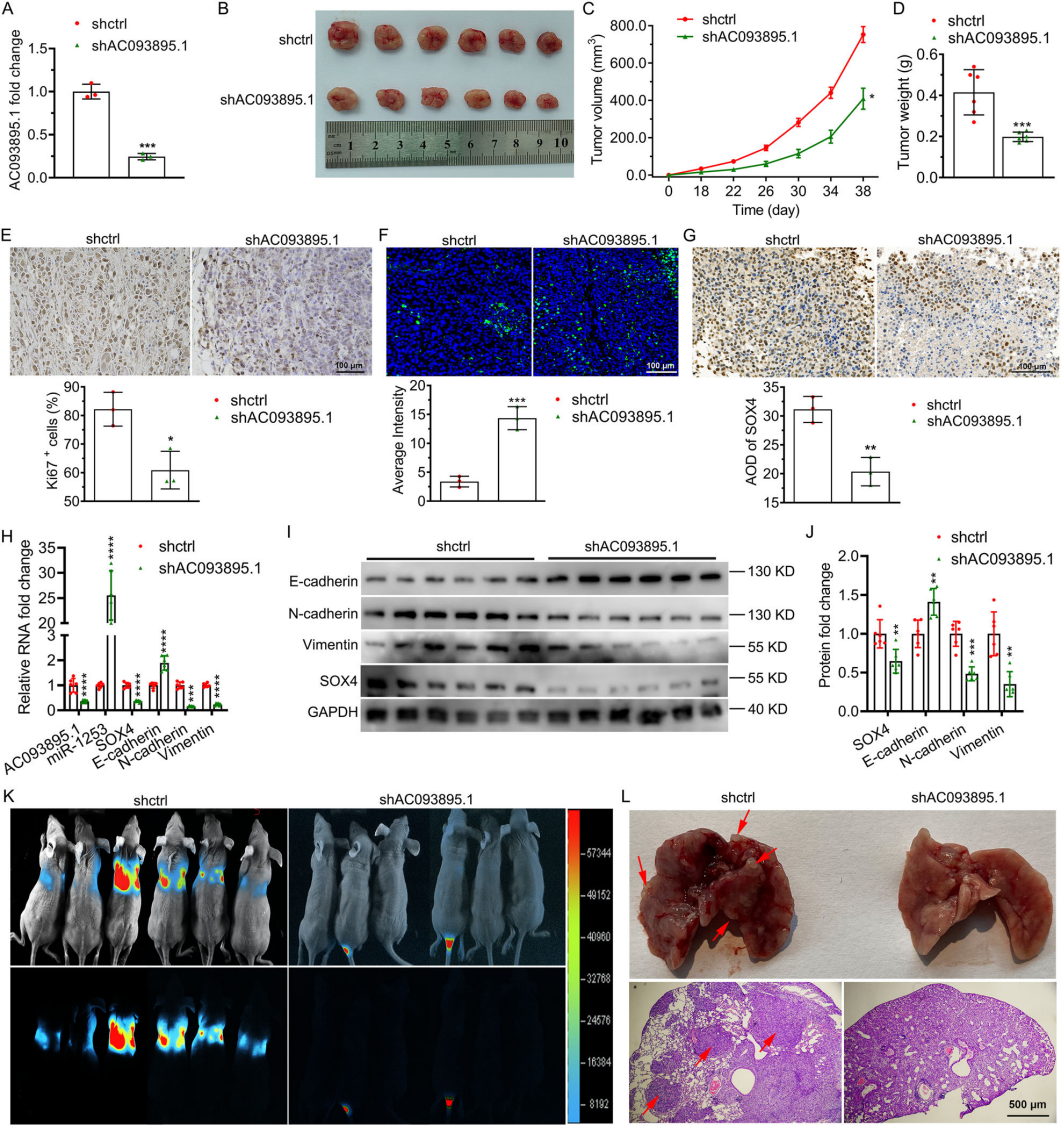
Depletion of AC093895.1 suppressed ovarian cancer cell growth and metastasis in mice and blocked the SOX4/AC093895.1/miR-1253/SOX4 loop. **A** AC093895.1 levels in SK-OV-3 cells with AC093895.1 knockdown were detected via RT-qPCR (*n* = 3/group). **B** Representative images of tumors formed in mice after subcutaneous injection of SK-OV-3 cells with AC093895.1 knockdown (*n* = 6/group). Volume (**C**) and quantified weight (**D**) of tumors formed from AC093895.1-depleted SK-OV-3 cells in mice (*n* = 6/group). Proliferation and apoptosis of tumor cells detected by immunohistochemical staining for Ki67 (**E**) and TUNEL immunofluorescence staining (**F**) (*n* = 3/group). **G** SOX4 expression in tumor tissues detected by immunohistochemistry. Quantification of AC093895.1, miR-1253, SOX4, or EMT markers by RT-qPCR (**H**) and western blotting (**I**, **J**) in tumors formed by AC093895.1-depleted cells (*n* = 6/group). **K** Representative images of mouse and lung metastases formed by tail vein-injected SK-OV-3 cells with AC093895.1 knockdown (*n* = 6/group). **L** Lung tissue from mice injected with SK-OV-3 cells via the tail vein (top) and hematoxylin and eosin staining of the lung tissue (bottom) (*n* = 3/group). Student’s *t* test was used to assess significant differences: *P < 0.05, **P < 0.01, ***P < 0.001.

SK-OV-3 cells expressing or lacking AC093895.1 were subcutaneously injected into the mice. Tumor weight and growth were significantly decreased following AC093895.1 knockdown (Fig. 8A–D). Moreover, in tumors formed by AC093895.1-knockdown cells, the expression of Ki67 was decreased, whereas TUNEL assays revealed increased apoptosis (Fig. 8E, F). Additionally, tumors in the shAC093895.1 group presented upregulation of miR-1253 and E-cadherin and downregulation of SOX4, vimentin, and N-cadherin (Fig. 8G–J). Furthermore, AC093895.1 knockdown attenuated lung metastasis in the pulmonary metastatic model established via tail vein injection of AC093895.1-depleted SK-OV-3 cells (Fig. 8K, L). These results suggest that AC093895.1 knockdown inhibits tumor growth and metastasis in mice, promotes miR-1253 expression, and inhibits SOX4 expression.

## Discussion and conclusion

As one of the most malignant tumors in women, OC not only affects fertility but also influences patients’ overall health [31]. The main challenge in OC treatment is tumor metastasis, particularly lung metastasis, which is the major cause of high mortality [32]. The complexity of OC regulation highlights the critical need to identify key targets or networks directly responsible for tumor progression and metastasis, which is essential for prognostic evaluation, clinical decision-making, and treatment development. Currently, several biomarkers such as CA125 and HE4 have been applied in clinical practice for ovarian cancer detection [33]. However, single biomarkers exhibit limitations in sensitivity and specificity, necessitating comprehensive evaluation with imaging and disease outcomes. Several potential OC biomarkers have been reported in recent studies. For example, the expression of SOX4 is increased in OC patients and is significantly correlated with FIGO staging, CA125 levels, lymph node status, and tumor size [34–36]. Notably, the tumor suppressor miR-1253 and its associated circRNA show superior diagnostic performance for OC compared to conventional biomarkers like CA125, CA153, and CEA [37, 38]. Therefore, these findings underscore the necessity for continued investigation and refinement of OC biomarkers and multimarker panels.

LncRNAs are a class of noncoding transcripts that play heterogeneous roles and constitute complex gene regulatory networks in tumorigenesis [5, 8, 39]. The targeting of lncRNAs is considered an advantageous alternative to conventional protein-based targets (e.g., phosphorylases, structural proteins) in cancer treatment, largely due to its association with fewer toxic side effects [13–16]. In this study, we identified AC093895.1 as a novel oncogenic lncRNA in OC. To address the common challenge of obtaining normal ovarian tissue controls, we leveraged publicly available datasets to validate our findings. Its elevated expression is correlated with poor prognosis and metastatic progression, positioning it as a robust prognostic indicator and a potential driver of aggressive disease.

LncRNAs can function as ceRNAs, facilitating with the ability to influence transcription and protein expression by interacting with miRNAs [19, 40, 41]. In our study, AC093895.1 was predominantly localized in cytoplasm, suggesting its potential role in miRNAs interference. Through bioinformatic analysis, we confirmed that AC093895.1 binds to miR-1253. miR-1253 has been established as a tumor suppressor in various cancers, including pancreatic ductal adenocarcinoma, prostate cancer, NSCLC, and osteosarcoma [19, 27, 42, 43]. However, its function in OC had remained unexplored. Our study revealed that miR-1253 expression is low in OC tissues and cell lines and that AC093895.1 directly regulates the tumor-suppressing effects of miR-1253 on OC.

Further, we performed transcriptome sequencing of A2780 and SK-OV-3 cells with AC093895.1 knockdown or miR-1253 inhibition to screen the key DEGs of downstream genes. Combined with the intersection analysis of DEGs and the bioinformatics results, SOX4, SEMA7A, and PPP1CC were identified as the downstream genes for both AC093895.1 and miR-1253. Among which, high expression of SEMA7A leads to drug resistance in primary tumors and promotes lung metastasis [44], while PPP1CC significantly promotes OC progression and migration [45]. In contrast, SOX4 functions as a transcription factor and an oncogene in diverse cancers [29, 46] and is directly associated with poor prognosis in prostate cancer patients [47]. Furthermore, the average expression of SOX4 in OC tissues was higher than that in normal tissues compared with SEMA7A and PPP1CC. Therefore, we pinpointed the transcription factor SOX4 as a critical downstream effector for further investigation.

The significance of focusing on SOX4 is twofold. First, we validated it as a direct functional target of miR-1253 in OC, a relationship confirmed by dual-luciferase assays and functional studies. Second, and more importantly, given that SOX4, as a transcription factor, can also regulate the levels of some lncRNAs [48], we discovered that it reciprocally binds to the promoter of AC093895.1. This interaction completes a previously unrecognized positive feedback loop: SOX4/AC093895.1/miR-1253/SOX4. This self-reinforcing circuit offers a powerful mechanistic explanation for the sustained high expression of both AC093895.1 and SOX4 in tumors, and the concurrent silencing of miR-1253.

In this study, we configured a novel SOX4/AC093895.1/miR-1253/SOX4 circular axis of molecular mechanisms that regulate the proliferation and metastatic capacity of OC cells. Correlation of gene expression within the loop was established in clinical OC samples via in situ multicolor IF, confirming the presence of this specific loop in OC tissues. The SOX4/AC093895.1/miR-1253/SOX4 loop may cause a cascade of amplifications, leading to increased expression of specific nodes, such as AC093895.1 and SOX4, which could further augment the expression of these oncogenic genes through interconnected regulatory mechanisms within the loop. In contrast, the loop profoundly suppressed the expression of the tumor suppressor gene miR-1253. Furthermore, analysis of clinical samples revealed that the activity of this SOX4/AC093895.1/miR-1253 loop is associated with advanced TNM stages in OC patients. This strong clinical correlation suggests that measuring the expression levels of this loop’s components could serve as an effective prognostic biomarker.

Given that SOX4 is associated with EMT in lung cancer and promotes cell migration [49] and tumor growth in pancreatic cancer [50], we evaluated whether it contributes to the high mortality of OC by inducing lung metastasis. Our in vivo experiments confirmed this by demonstrating that knocking down AC093895.1—a pro-cancer node within the loop—effectively suppressed tumor growth and lung metastasis. This anti-tumor effect was associated with the disruption of the feedback mechanism, evidenced by the restoration of miR-1253 expression and the downregulation of SOX4. This positions the AC093895.1 node and the feedback loop itself as promising therapeutic targets, particularly for metastatic OC.

In conclusion, our study moves beyond the identification of another dysregulated lncRNA in cancer. We elucidate a novel, self-sustaining oncogenic circuit centered on the SOX4/AC093895.1/miR-1253/SOX4 loop that drives OC progression and metastasis. This model not only provides a fresh perspective on the molecular pathogenesis of OC but also unveils a prognostic biomarker panel and a vulnerable regulatory axis with significant potential for therapeutic intervention against advanced and metastatic disease.

## Materials and methods

### Bioinformatic analysis

Transcriptome profiling datasets of 422 patients with OC and 193 healthy individuals were downloaded from the GTEx [51] and TCGA databases [52]. GraphPad Prism software (version 9.5) was used to detect the differential expression of AC093895.1 in healthy and tumor tissues at different clinical stages. Patients with OC were divided into two subgroups.

The miRDB, lncBase, and lncRNASNP2 databases were used to identify miRNAs that could directly interact with AC093895.1. The miRTarBase, TargetScan, MicroT, and miRDB databases were used to screen for downstream target genes that could directly interact with miR-1253.

### Tissue microarray analysis

OC tissue microarray (HOvaC151Su02) samples were purchased from Shanghai Outdo Biotech Company. The tissue sections were scanned via a PANORAMIC panoramic section scanner, and the number of positive or total cells in the destination area of each chip was calculated separately via Halo v3.0.311.314 analysis software to determine the rate of positivity (%). Analysis of the differential expression of AC093895.1 in primary tumors and metastatic tumors using the tissue microarray was approved by the Ethics Committee of Shanghai Outdo Biotech Company (SHYJS-CP-1804029).

### Cell lines

All cell lines were purchased in 2019 from Xiamen Immocell Biotechnology Co., Ltd. (http://www.immocell.com) and cultured under standardized conditions: Immortalized Ovarian Surface Epithelial Cell Line 80 (IOSE-80) (Catalog No. IM-H366, RRID: CVCL_5546), Human Ovarian Carcinoma Cell Line A2780 (Catalog No. IM-H002, RRID: CVCL_0134), Human Ovarian Cancer Cell Line CoC1 (Catalog No. IM-H006, RRID: CVCL_6891), and Human Ovarian Clear Cell Carcinoma Cells ES-2 (Catalog No. IM-H007, RRID: CVCL_3509) were maintained in RPMI-1640 medium (Immocell, IMC-202) supplemented with 10% fetal bovine serum (FBS; Immocell, IMC-101) and 1% penicillin–streptomycin solution (Immocell, IMC-601). Human Ovarian Carcinoma Cell Line HEY (Catalog No. IM-H419, RRID: CVCL_0297) was cultured in DMEM (Immocell, IMC-201). Human Ovarian Carcinoma Cell Line SK-OV-3 (Catalog No. IM-H005, RRID: CVCL_0532) was cultured in McCoy’s 5 A (modified) medium (Gibco, 16600108). All media were supplemented with 10% FBS and 1% penicillin–streptomycin. Cells were incubated at 37 °C in a humidified atmosphere. Prior to experiments, all cell lines underwent authentication via short tandem repeat (STR) profiling and were confirmed mycoplasma-free using a Mycoplasma PCR Detection Kit (Beyotime, C0301S).

### Dual-luciferase reporter assays (Dual-LUC)

A PGL4.20 and pmirGLO vector (Anti-HeLa, Xiamen, China) was used to insert wild-type and mutant fragments of the AC093895.1 or SOX4 3’ UTR to generate the corresponding constructs. A2780 and SK-OV-3 cells were transfected with the indicated plasmids via Lipofectamine 2000 (Invitrogen, 11668-019, China) in 24-well plates. After 48 h of transfection, AC093895.1 or SOX4 3’ UTR activity was determined via dual-luciferase reporter analysis (Promega, E1910). Relative luciferase activity was normalized to Renilla luciferase activity, and each group was replicated three times.

### RNA pull-down

To identify the potential binding between AC093895.1 and miR-1253, RNA pull-down assays were performed. Briefly, cell lysates from A2780 and SK-OV-3 cells were incubated with biotin-labeled wild-type (WT) or mutant (MUT) miR-1253 probes. The RNA complexes were isolated using streptavidin-coated magnetic beads. Subsequently, the enrichment of AC093895.1 in the pull-down material was detected by RT-qPCR and visualized via agarose gel electrophoresis.

### Generation of AC093895.1-knockdown cell lines

Lentiviral knockdown plasmids (Anti-HeLa, Xiamen, China) were packaged into lentiviruses as previously described [53]. Briefly, A2780 and SK-OV-3 cells were treated with lentivirus-containing medium. Puromycin (1.0 μg/mL) was added after 48 h, and AC093895.1 expression was detected at 72 h after infection.

### Transfection of miR-1253 mimic or inhibitor and shSOX4 or SOX4-overexpressing plasmids

A2780 and SK-OV-3 cells were seeded and transfected with the miR-1253 mimic, negative control (NC) mimic, NC inhibitor, miR-1253 inhibitors (RiboBio, Guangzhou, China), shSOX4, or plasmids expressing SOX4 (pCDH-EF1α-MCS-T2A-Puro vector, without its 3′UTR, Anti-HeLa, Xiamen, China) via Lipofectamine 2 000 reagent.

### Cell proliferation assays

The cells were seeded in 96-well plates, and MTT assays were performed by subjecting the cells to the MTT reagent (1 mg/mL) after 1, 2, or 3 d of transfection. The culture supernatant was replaced with DMSO after 4 h of incubation, and the OD490 value was subsequently quantified via an absorbance reader (Molecular Devices, San Francisco, CA, USA). Additionally, an EdU assay (RiboBio, Guangzhou, China) was performed 24 h after seeding. Representative images were captured via a fluorescence microscope (Motic, China).

### Cell cycle and apoptosis quantification

Following 48 h of transfection or lentivirus infection, propidium iodide (ST511; Beyotime) was used to analyze the different cell cycle phases in the collected cells and to perform apoptotic analysis via an Annexin V-FITC Kit (Beyotime, C1062M), as described previously.

### Wound healing assays

A2780 and SK-OV-3 cells expressing short hairpin RNA targeting AC093895.1 (shAC093895.1) and NC cells were seeded onto 6-well plates. A linear wound of a specific size was created by scratching the culture plate according to the experimental plan. The cells were continuously cultured at 37 °C in basic culture medium for 48 h. During the culture process, the morphological changes in the cells were observed via an optical microscope (Motic incorporation; × 40). The photos were then input into ImageJ 1.8.0 software for automatic processing, the wound healing ratio was calculated, and the results were compared [54].

### Migration and invasion analyses

To minimize the impact of cell proliferation on the assessment of migratory and invasive capabilities, the following protocol was employed. Cells in good growth condition were starved in serum-free medium for 24 h. Subsequently, the treated cells were harvested, digested, and resuspended in serum-free medium to adjust the cell density to an appropriate concentration. After 48 h of seeding, cell migration and invasion were assessed using Transwell plates with or without Matrigel coating.

### RNA-seq transcriptomics analysis

A2780 and SK-OV-3 cells with AC093895.1 knockdown or miR-1253 inhibition were analyzed via transcriptome sequencing via the Illumina system. Genes with a ∣log2∣fold change ≥ 1 and *P* < 0.05 were defined and screened as significantly DEGs. The intersection of these genes was then analyzed to identify the genes downstream of both AC093895.1 and miR-1253.

### Immunohistochemistry analysis

After the isolated tumors were sectioned, the paraffin was removed, and peroxidase activity was inhibited. Following antigen retrieval, a primary antibody against Ki67 (proteintech, 28074-1-AP, 1:100 dilution) or SOX4 (HUABIO, ER1916-97, 1:100 dilution) was applied, and the slides were cultured for 16 h at 4 °C. The samples were subsequently incubated with the corresponding secondary antibody (HUABIO, HA1119) for 0.5 h at room temperature, followed by treatment with the Vectastain complex (Vector Laboratories, PK-6100) for 0.5 h. The slides were developed with DAB reagent (MXB Biotechnologies, DAB-0031), counterstained with Mayer’s hematoxylin solution (Sigma, MHS80) for 1 min, and dehydrated. The slides were mounted with Entellan mounting medium (Merck, 107961).

### Chromatin immunoprecipitation assays

The interaction between SOX4 and the AC093895.1 promoter was assessed via ChIP [30]. Briefly, 1% formaldehyde diluted in PBS was applied to the crosslinked cells at 25 °C for 10 min, and the resulting cells were quenched by direct exposure to 1.275 M glycine. Prior to centrifugation at 300 × *g* for 5 min, the fixed cells were lysed in a swelling buffer for 10 min on ice. The pellet was sonicated for 20 s and centrifuged at 14,000 rpm for 15 min. Control IgG (2 μg) or anti-SOX4 antibody (ab86809; Abcam) was added, and the mixture was cultured at 4 °C for 2 h. Protein G Sepharose (40 µL) beads were used to capture the antibodies with rotation at 4 °C for 12 h, which were subsequently washed three times, and the RNA was removed. The DNA was subsequently precipitated and dissolved in 100 µL of Tris buffer (pH 7.5). PCR was performed to measure the DNA fragment levels.

### In situ hybridization

A2780 and SK-OV-3 cells (3 × 10^5^) were cultured in 6-well plates with glass slides for 24 h. A fluorescent hybridization kit (RiboBio, C10910) was used to measure the localization of AC093895.1, 18S rRNA, and U6 snRNA, according to the manufacturer’s instructions. A laser confocal microscope (Leica) was used to capture representative images.

### Immunofluorescence and fluorescence in situ hybridization (FISH)

Combined fluorescence in situ hybridization (FISH) and multiplex immunofluorescence (mIF) were performed on paraffin-embedded tissue sections. Following deparaffinization and antigen retrieval, sections were subjected to pre-hybridization, hybridized with specific probes overnight, and stringently washed. Subsequently, multiplex immunofluorescence staining was carried out using a standardized protocol involving sequential incubation with primary antibodies, HRP-conjugated secondary antibodies, and tyramide signal amplification with fluorophores (520 nm, 570 nm, 620 nm, 690 nm). Nuclei were counterstained with DAPI. Images were captured via a Nikon DS-U3 system. The differential expression of AC093895.1, miR-1253 and SOX4 in ovarian cancer was analyzed by using tissue sections and approved by the Life Sciences Ethics Committee of Changsha Yaxiang Biotechnology Co., LTD. (CSYAYJ2024026).

### Animal experiments

The Fujian University of Traditional Chinese Medicine Laboratory Animal Center provided 24 5 to 6-week-old BALB/c nude mice for the experiments. For the tumor growth assays, 4 × 10^6^ cells stably expressing shAC093895.1 or the NC were inoculated subcutaneously into the left chest of the mice (*n* = 6 per group). The tumor volume was measured every 4 days for 18 days post-inoculation. At 38 days post-inoculation, the mice were euthanized via an intraperitoneal injection of pentobarbital sodium (150 mg/kg). After confirming cardiac and respiratory arrest, along with the absence of neural reflexes, the tissues were excised for subsequent investigation. The tumors were excised and weighed. For the pulmonary metastatic model, 4 × 10^6^ cells stably expressing luciferase and shAC093895.1 or NC cells were injected into the tail veins of the mice (*n* = 6 per group). Pulmonary metastasis was detected 42 days postinjection via a Lumina II system (Xenogen, Germany). The animal experimental procedures were performed in accordance with the Guidelines for the Care and Use of Laboratory Animals formulated by the National Institutes of Health, and the protocols were approved by the Medical Ethics Committee of Fujian University of Traditional Chinese Medicine (Approval no. FJTCM IACUC 2024017).

### HE staining analysis

Murine lung tissues were embedded in paraffin. After dewaxing, the sections were stained with Mayer’s hematoxylin and 1% water-soluble eosin and magnified under a microscope.

### TUNEL detection assays

The samples were embedded in paraffin and sectioned. After dewaxing, the sections were stained with an apoptosis detection kit (Shanghai Yisheng, 40308ES20, China) and DAPI (Beyotime, C1002, China) and photographed.

### Statistical analysis

Two-group and multiple-group comparisons were performed via a separate t-test and ANOVA, respectively, via SPSS 22.0. Differences were considered statistically significant at *P* < 0.05 (*0.01 < *P* < 0.05; **0.001 < *P* < 0.01; ***P < 0.001). Graphs were generated via GraphPad software version 8.2.1.

## Supplementary information

Supplementary Figure

Supplementary Tables S1

Supplementary Tables S2

Supplementary Tables S3

original western blots

## Data Availability

All data needed to evaluate the conclusions in the paper are present in the paper and/or the Supplementary Materials.

## References

[1] Bray F, Laversanne M, Sung H, Ferlay J, Siegel RL, Soerjomataram I, et al. Global cancer statistics 2022: GLOBOCAN estimates of incidence and mortality worldwide for 36 cancers in 185 countries. CA Cancer J Clin. 2024;74:229–63. 38572751 10.3322/caac.21834

[2] Hao L, Boehnke N, Elledge SK, Harzallah NS, Zhao RT, Cai E, et al. Targeting and monitoring ovarian cancer invasion with an RNAi and peptide delivery system. Proc Natl Acad Sci USA. 2024;121:e2307802121. 38437557 10.1073/pnas.2307802121 PMC10945808

[3] Gong L, Li G, Yi X, Han Q, Wu Q, Ying F, et al. Tumor-derived small extracellular vesicles facilitate omental metastasis of ovarian cancer by triggering activation of mesenchymal stem cells. Cell Commun Signal. 2024;22:47. 38233863 10.1186/s12964-023-01413-9 PMC10795335

[4] Giampaolino P, Foreste V, Della Corte L, Di Filippo C, Iorio G, Bifulco G. Role of biomarkers for early detection of ovarian cancer recurrence. Gland Surg. 2020;9:1102–11. 32953625 10.21037/gs-20-544 PMC7475347

[5] Zou Y, Gao B, Lu J, Zhang K, Zhai M, Yuan Z, et al. Long non-coding RNA CASC15 enhances learning and memory in mice by promoting synaptic plasticity in hippocampal neurons. Exploration. 2024;4:20230154. 10.1002/EXP.20230154 PMC11655312 39713210

[6] Coan M, Haefliger S, Ounzain S, Johnson R. Targeting and engineering long non-coding RNAs for cancer therapy. Nat Rev Genet. 2024;25:578–95. 38424237 10.1038/s41576-024-00693-2

[7] Nadhan R, Isidoro C, Song YS, Dhanasekaran DN. LncRNAs and the cancer epigenome: mechanisms and therapeutic potential. Cancer Lett. 2024;605:217297. 39424260 10.1016/j.canlet.2024.217297

[8] Li Y, Li F, Ding M, Ma Z, Li S, Qu J, et al. Chuanxiong Rhizoma extracts prevent liver fibrosis via targeting CTCF-c-MYC-H19 pathway. Chin Herb Med. 2024;16:82–93. 38375042 10.1016/j.chmed.2023.07.003 PMC10874761

[9] Tang J, Wang X, Xiao D, Liu S, Tao Y. The chromatin-associated RNAs in gene regulation and cancer. Mol Cancer. 2023;22:27. 36750826 10.1186/s12943-023-01724-y PMC9903551

[10] Ha JH, Radhakrishnan R, Nadhan R, Gomathinayagam R, Jayaraman M, Yan M, et al. Deciphering a GPCR-lncRNA-miRNA nexus: identification of an aberrant therapeutic target in ovarian cancer. Cancer Lett. 2024;591:216891. 38642607 10.1016/j.canlet.2024.216891 PMC12927523

[11] Dubiez E, Pellegrini E, Finderup Brask M, Garland W, Foucher AE, Huard K, et al. Structural basis for competitive binding of productive and degradative co-transcriptional effectors to the nuclear cap-binding complex. Cell Rep. 2024;43:113639. 38175753 10.1016/j.celrep.2023.113639

[12] Song Y, Chen C, Li W. Ginsenoside Rb1 in cardiovascular and cerebrovascular diseases: a review of therapeutic potentials and molecular mechanisms. Chin Herb Med. 2024;16:489–504. 39606264 10.1016/j.chmed.2024.09.006 PMC11589305

[13] Zhang R, Yang R, Huang Z, Xu X, Lv S, Guan X, et al. METTL3/YTHDC1-mediated upregulation of LINC00294 promotes hepatocellular carcinoma progression. Heliyon. 2023;9:e22595. 38125436 10.1016/j.heliyon.2023.e22595 PMC10730722

[14] Jiang T, Wang Y, Chen X, Xia W, Xue S, Gu L, et al. Neutrophil extracellular traps (NETs)-related lncRNAs signature for predicting prognosis and the immune microenvironment in breast cancer. Front Cell Dev Biol. 2023;11:1117637. 36819091 10.3389/fcell.2023.1117637 PMC9932980

[15] Li P, Ma X, Gu X. LncRNA MAFG-AS1 is involved in human cancer progression. Eur J Med Res. 2023;28:497. 37941063 10.1186/s40001-023-01486-9 PMC10631199

[16] Khorkova O, Stahl J, Joji A, Volmar CH, Wahlestedt C. Amplifying gene expression with RNA-targeted therapeutics. Nat Rev Drug Discov. 2023;22:539–61. 37253858 10.1038/s41573-023-00704-7 PMC10227815

[17] Abd-Allah GM, Ismail A, El-Mahdy HA, Elsakka EGE, El-Husseiny AA, Abdelmaksoud NM, et al. miRNAs as potential game-changers in melanoma: a comprehensive review. Pathol Res Pract. 2023;244:154424. 36989843 10.1016/j.prp.2023.154424

[18] Azizi M, Salehi-Mazandarani S, Nikpour P, Andalib A, Rezaei M. The role of unfolded protein response-associated miRNAs in immunogenic cell death amplification: a literature review and bioinformatics analysis. Life Sci. 2023;314:121341. 36586572 10.1016/j.lfs.2022.121341

[19] Chen Y, Gu M, Liu C, Wan X, Shi Q, Chen Q, et al. Long noncoding RNA FOXC2-AS1 facilitates the proliferation and progression of prostate cancer via targeting miR-1253/EZH2. Gene. 2019;686:37–42. 30389560 10.1016/j.gene.2018.10.085

[20] Xu Y, Yao Y, Gao P, Cui Y. Upregulated circular RNA circ_0030235 predicts unfavorable prognosis in pancreatic ductal adenocarcinoma and facilitates cell progression by sponging miR-1253 and miR-1294. Biochem Biophys Res Commun. 2019;509:138–42. 30591218 10.1016/j.bbrc.2018.12.088

[21] Huang L, Chen M, Pan J, Yu W. Circular RNA circNASP modulates the malignant behaviors in osteosarcoma via miR-1253/FOXF1 pathway. Biochem Biophys Res Commun. 2018;500:511–7. 29678578 10.1016/j.bbrc.2018.04.131

[22] Liu M, Zhang Y, Zhang J, Cai H, Zhang C, Yang Z, et al. MicroRNA-1253 suppresses cell proliferation and invasion of non-small-cell lung carcinoma by targeting WNT5A. Cell Death Dis. 2018;9:189. 29415994 10.1038/s41419-017-0218-x PMC5833797

[23] Wen T, Zhang X, Gao Y, Tian H, Fan L, Yang P. SOX4-BMI1 axis promotes non-small cell lung cancer progression and facilitates angiogenesis by suppressing ZNF24. Cell Death Dis. 2024;15:698. 39349443 10.1038/s41419-024-07075-w PMC11442842

[24] Zhao S, Li P, Zhou G. Long noncoding RNAs in the prediction of survival of patients with digestive cancers. Turk J Gastroenterol. 2023;34:19–25. 36445051 10.5152/tjg.2022.22017 PMC9984979

[25] Duan WW, Yang LT, Liu J, Dai ZY, Wang ZY, Zhang H, et al. A TGF-beta signaling-related lncRNA signature for prediction of glioma prognosis, immune microenvironment, and immunotherapy response. CNS Neurosci Ther. 2024;30:e14489. 37850692 10.1111/cns.14489 PMC11017415

[26] Yang D, Zhang D. miR-1253, a novel tumor suppressor gene in colon cancer, is associated with poor patient prognosis. Clin Exp Med. 2021;21:563–71. 33837882 10.1007/s10238-021-00706-y

[27] Xiong W, Yang J. CircSEC24A induces KLF8 expression to promote the malignant progression of non-small cell lung cancer by regulating miR-1253. Thorac Cancer. 2024;15:2461–73. 10.1111/1759-7714.15450 PMC11646692 39465973

[28] Ding X, Zheng J, Cao M. Circ_0004771 accelerates cell carcinogenic phenotypes via suppressing miR-1253-mediated DDAH1 inhibition in breast cancer. Cancer Manag Res. 2021;13:1–11. 33442289 10.2147/CMAR.S273783 PMC7797298

[29] Sheykhi-Sabzehpoush M, Ghasemian M, Khojasteh Pour F, Mighani M, Moghanibashi M, Mohammad Jafari R, et al. Emerging roles of long non-coding RNA FTX in human disorders. Clin Transl Oncol. 2023;25:2812–31. 37095425 10.1007/s12094-023-03163-z

[30] Pramio D, Schechtman D. Chromatin immunoprecipitation on fixed tissues and cell lines. Methods Mol Biol. 2023;2599:21–31. 36427140 10.1007/978-1-0716-2847-8_3

[31] Shahid S, Khan A, Shahid W, Rehan M, Asif R, Nisar H, et al. Nanoenzymes: a radiant hope for the early diagnosis and effective treatment of breast and ovarian cancers. Int J Nanomed. 2024;19:5813–35. 10.2147/IJN.S460712 PMC11184228 38895143

[32] Qian L, Chen Y, Peng M, Xia Y, Zhou T, Hong J, et al. The importance of marital status in the morbidity and prognosis of lung metastasis in newly diagnosed ovarian cancer. J Cancer. 2023;14:1024–38. 37151400 10.7150/jca.83017 PMC10158508

[33] Drymiotou S, Theodorou E, Rallis KS, Nicolaides M, Sideris M. Molecular biomarkers in borderline ovarian tumors: towards personalized treatment and prognostic assessment. Cancers. 2025;17:545. 10.3390/cancers17030545 PMC11816664 39941911

[34] Ren Z, Hu Y, Chen J, & Jin L. MicroRNA-200a inhibits TGF-β-induced epithelial-mesenchymal transition in human ovarian carcinoma cells by downregulating SOX4 expression. Arch Med Sci. 2021. 10.5114/aoms/139106.

[35] Xi J, Feng J, Zeng S. Long noncoding RNA lncBRM facilitates the proliferation, migration and invasion of ovarian cancer cells via upregulation of Sox4. Am J Cancer Res. 2017;7:2180–9. 29218242 PMC5714747

[36] Yeh YM, Chuang CM, Chao KC, Wang LH. MicroRNA-138 suppresses ovarian cancer cell invasion and metastasis by targeting SOX4 and HIF-1α. Int J Cancer. 2013;133:867–78. 23389731 10.1002/ijc.28086

[37] Loric S, Denis JA, Desbene C, Sabbah M, Conti M. Extracellular vesicles in breast cancer: from biology and function to clinical diagnosis and therapeutic management. Int J Mol Sci. 2023;24:7208. 10.3390/ijms24087208 PMC10139222 37108371

[38] Buczyńska A, Sidorkiewicz I, Niemira M, Krętowski AJ, Węgrzyn P, Kosiński P, et al. Identification of MicroRNA profiles in fetal spina bifida: the role in pathomechanism and diagnostic significance. Int J Mol Sci. 2024;25:2896. 10.3390/ijms25052896 PMC10931641 38474143

[39] Feng D, Li D, Wang J, Wu R, Zhang C. Senescence-associated lncRNAs indicate distinct molecular subtypes associated with prognosis and androgen response in patients with prostate cancer. Acta Mater Med. 2023;2:299–309.

[40] Li J, Li Z, Wang Y, Lin H, Wu B. TLSEA: a tool for lncRNA set enrichment analysis based on multi-source heterogeneous information fusion. Front Genet. 2023;14:1181391. 37205123 10.3389/fgene.2023.1181391 PMC10185877

[41] Liu J, Zhang B, Wang L, Li S, Long Q, Xiao X. Bioactive components, pharmacological properties and underlying mechanism of Ganoderma lucidum spore oil: a review. Chin Herb Med. 2024;16:375–91. 39072196 10.1016/j.chmed.2023.09.007 PMC11283234

[42] He H, Li T. Hsa_circ_0000190 promotes NSCLC cell resistance to cisplatin via the modulation of the miR-1253/IL-6 Axis. Anal Cell Pathol. 2024;2024:6647810. 10.1155/2024/6647810 PMC10911877 38440120

[43] Mo J, Zheng T, Lei L, Dai P, Liu J, He H, et al. MicroRNA-1253 suppresses cell proliferation, migration, and invasion of osteosarcoma by targeting MMP9. Technol Cancer Res Treat. 2021;20:1533033821995278. 34036868 10.1177/1533033821995278 PMC8161890

[44] Crump LS, Wyatt GL, Rutherford TR, Richer JK, Porter WW, Lyons TR. Hormonal regulation of semaphorin 7a in ER(+) breast cancer drives therapeutic resistance. Cancer Res. 2021;81:187–98. 33122307 10.1158/0008-5472.CAN-20-1601 PMC7878309

[45] Feng P, Wang Y, Liu N, Chen Y, Hu Y, Huang Z, et al. High expression of PPP1CC promotes NHEJ-mediated DNA repair, leading to radioresistance and poor prognosis in nasopharyngeal carcinoma. Cell Death Differ. 2024;31:683–96. 38589496 10.1038/s41418-024-01287-5 PMC11094031

[46] Zhai Y, Zhang F, Zhou J, et al. Mechanism of norcantharidin intervention in gastric cancer: analysis based on antitumor proprietary Chinese medicine database, network pharmacology, and transcriptomics. Chin Med. 2024;19:129. 39289763 10.1186/s13020-024-01000-1 PMC11406961

[47] Li Z, Wang F. Integrative analysis of the SOX family-related prognostic signature and immunological infiltration in prostate cancer. Transl Cancer Res. 2023;12:2048–62. 37701109 10.21037/tcr-23-501 PMC10493808

[48] Wu D, He X, Wang W, Hu X, Wang K, Wang M. Long noncoding RNA SNHG12 induces proliferation, migration, epithelial-mesenchymal transition, and stemness of esophageal squamous cell carcinoma cells via post-transcriptional regulation of BMI1 and CTNNB1. Mol Oncol. 2020;14:2332–51. 32239639 10.1002/1878-0261.12683 PMC7463312

[49] Liu X, Wang Y, Zhou G, Zhou J, Tian Z, Xu J. circGRAMD1B contributes to migration, invasion and epithelial-mesenchymal transition of lung adenocarcinoma cells via modulating the expression of SOX4. Funct Integr Genom. 2023;23:75. 10.1007/s10142-023-00972-x 36867268

[50] Wang S, Zhu X, Hao Y, Su TT, Shi W. ALKBH5-mediated m6A modification of circFOXP1 promotes gastric cancer progression by regulating SOX4 expression and sponging miR-338-3p. Commun Biol. 2024;7:565. 38745044 10.1038/s42003-024-06274-7 PMC11094028

[51] Consortium GT. The genotype-tissue expression (GTEx) project. Nat Genet. 2013;45:580–5. 23715323 10.1038/ng.2653 PMC4010069

[52] Li J, Ma S, Zheng Y, Qin M, Jia H, Liu C, et al. Prognostic value and immune infiltration analyses of cuproptosis-related genes in hepatocellular carcinoma. Acta Mater Med. 2023;2:386–99.

[53] Zhang X, Wang C, Xu H, Cai S, Liu K, Li S, et al. Propofol inhibits myocardial injury induced by microvesicles derived from hypoxia-reoxygenated endothelial cells via lncCCT4-2/CCT4 signaling. Biol Res. 2023;56:20. 37143143 10.1186/s40659-023-00428-3 PMC10161458

[54] Bai X, Kang J, Wei S, Wang Y, Liu Y, Yuan B, et al. A pH-responsive nanocomposite for combination sonodynamic-immunotherapy with ferroptosis and calcium ion overload via SLC7A11/ACSL4/LPCAT3 pathway. Exploration. 2024;5:20240002. 10.1002/EXP.20240002 PMC11875445 40040833

