# Supplementary material for: The long noncoding RNA AC093895.1 promotes ovarian cancer formation and metastasis through a positive feedback network dependent on the transcription factor SOX4
Source: Cell Death Dis. 2026 Feb 3;17(1):202. doi: 10.1038/s41419-026-08429-2 (PMC12894752; doi:10.1038/s41419-026-08429-2)
Supplement: Supplementary file 1 — Supplementary Figure [file 41419_2026_8429_MOESM1_ESM.docx]

**Supplementary Figure**

**
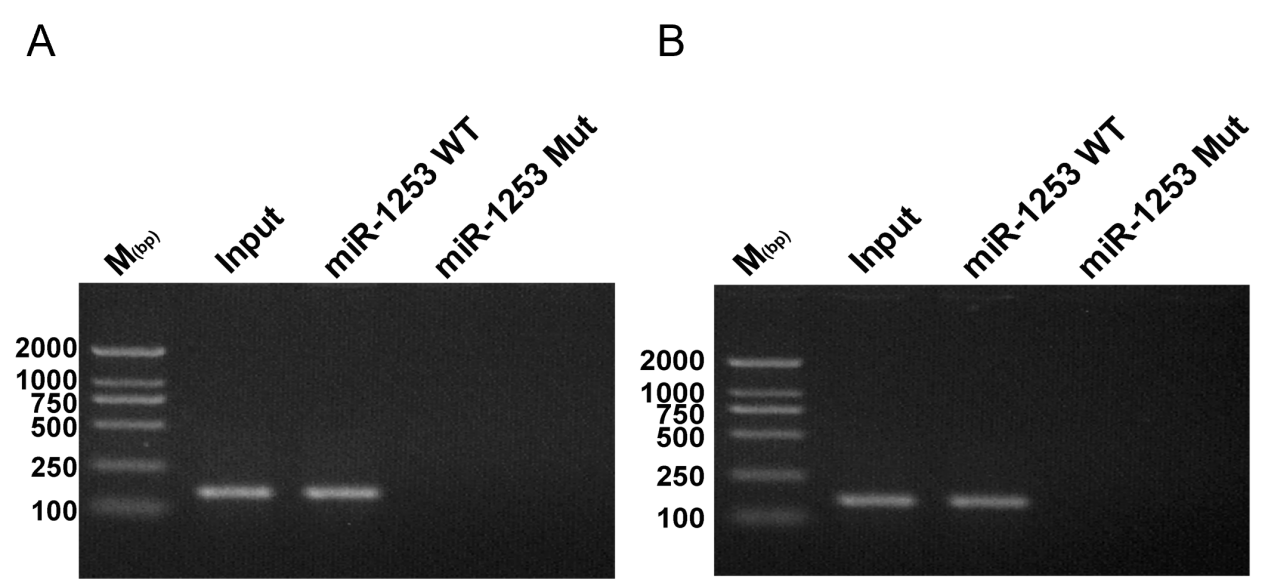
**

**Fig. S1 AC093895.1 directly binds to miR-1253 as determined by RNA pull-down assay.** Lysates from A2780 (A) and SKOV3 (B) cells were incubated with biotin-labeled wild-type (WT) or mutant (MUT) miR-1253 probes. The bound RNAs were pulled down using streptavidin magnetic beads and subsequently detected by RT-PCR and agarose gel electrophoresis.
