# Supplementary material for: The long noncoding RNA AC093895.1 promotes ovarian cancer formation and metastasis through a positive feedback network dependent on the transcription factor SOX4
Source: Cell Death Dis. 2026 Feb 3;17(1):202. doi: 10.1038/s41419-026-08429-2 (PMC12894752; doi:10.1038/s41419-026-08429-2)
Supplement: Supplementary file 2 — Supplementary Tables S1 [file 41419_2026_8429_MOESM2_ESM.docx]

Tables S1. Primers for plasmid construction and RT-qPCR

| **Primers** | | **Sequence (5’-3’)** |
| --- | --- | --- |
| shAC093895.1-1 | F | CCGGAGAAGTCTTCTCAAATGAATCCTCGAGGATTCATTTGAGAAGACTTCTTTTTT |
|  | R | AATTAAAAAAGAAGTCTTCTCAAATGAATCCTCGAGGATTCATTTGAGAAGACTTCT |
| shAC093895.1-2 | F | CCGGGAGTGAGCTTGGAAGCAGATGCTCGAGCATCTGCTTCCAAGCTCACTCTTTTT |
|  | R | AATTAAAAAGAGTGAGCTTGGAAGCAGATGCTCGAGCATCTGCTTCCAAGCTCACTC |
| SOX4 OE | F | CTAGAGCTAGCGAATTCGCCACCATATGGTGCAGCAAACCAACAATG |
|  | R | CAGCGGCCGCGGATCCGTAGGTGAAAACCAGGTTGG |
| Promoter WT-Luc | F | CTAACTGGCCGGTACCAGTCTAATTTGGGCTCCATTTAAC |
|  | R | TCTTGATATCCTCGAGCCACTAAGTTTTGGGATAATTTG |
| Promoter M1-Luc | F | TGGGAGACAGCACGCAAATTTTGACTAGCCTAATTTGG |
|  | R | AATTTGCGTGCTGTCTCCCAAATCACAGATGTTTTAATGC |
| Promoter M2-Luc | F | TCCACTCCACAGATCTCAAAATCCTTTTCTTTTTCTCC |
|  | R | TTTGAGATCTGTGGAGTGGAGGCATGAAGGAG |
| U6 | RT | AACGCTTCACGAATTTGCGT |
|  | QF | CTCGCTTCGGCAGCACA |
|  | QR | AACGCTTCACGAATTTGCGT |
| MiR-1253 | RT | GTCGTATCCAGTGCAGGGTCCGAGGTATTCGCACTGGATACGACTGCAGG |
|  | QF | CGCGCGAGAGAAGAAGATCAG |
|  | QR | AGTGCAGGGTCCGAGGTATT |
| 18S | QF | AGGCGCGCAAATTACCCAATCC |
|  | QR | GCCCTCCAATTGTTCCTCGTTAAG |
| AC093895.1 | QF | CGGTGGGAGGAACAGAATC |
|  | QR | CATAGGCTGCAATCAAGGTG |
| SOX4 | QF | GACATGCACAACGCCGAGATCT |
|  | QR | GTAGTCAGCCATGTGCTTGAGG |
| AC093895.1-SOX4 Target 1 | F | GGAATGTATAGGTACACTAC |
|  | R | CTGCTTCTCCCATCTGCATG |
| AC093895.1-SOX4  Target 2 | F | CTATTCCTTAAACACTGAG |
|  | R | AGCAGACTAGGATACAAGAC |
| SOX4 shRNA-1 | F | CCGGGGACAGACGAAGAGTTTAAAGCTCGAGCTTTAAACTCTTCGTCTGTCCTTTTT |
|  | R | AATTAAAAAGGACAGACGAAGAGTTTAAAGCTCGAGCTTTAAACTCTTCGTCTGTCC |
| SOX4 shRNA-2 | F | CCGGGGAGAGGGACTAAGTGCTAGACTCGAGTCTAGCACTTAGTCCCTCTCCTTTTT |
|  | R | AATTAAAAAGGAGAGGGACTAAGTGCTAGACTCGAGTCTAGCACTTAGTCCCTCTCC |
| SOX4 shRNA-3 | F | CCGGCCGACGACGAGTTCGAAGACGCTCGAGCGTCTTCGAACTCGTCGTCGGTTTTT |
|  | R | AATTAAAAACCGACGACGAGTTCGAAGACGCTCGAGCGTCTTCGAACTCGTCGTCGG |
