# Supplementary material for: The long noncoding RNA AC093895.1 promotes ovarian cancer formation and metastasis through a positive feedback network dependent on the transcription factor SOX4
Source: Cell Death Dis. 2026 Feb 3;17(1):202. doi: 10.1038/s41419-026-08429-2 (PMC12894752; doi:10.1038/s41419-026-08429-2)
Supplement: Supplementary file 3 — Supplementary Tables S2 [file 41419_2026_8429_MOESM3_ESM.docx]

**Table S2. Organ metastasis in 14 clinical cases of ovarian cancer patients**

| **metastatic focus**  **serial**  **number** | **Uterus** | **​​Peritoneum​** | **​​Colon** | **​​** **Diaphragm** |
| --- | --- | --- | --- | --- |
| **H1** | metastasis​ | metastasis​ | metastasis​ | metastasis​ |
| **H2** | non-metastatic​ | non-metastatic​ | metastasis​ | non-metastatic​ |
| **H3** | non-metastatic​ | non-metastatic​ | metastasis​ | non-metastatic |
| **H4** | metastasis​ | metastasis​ | metastasis​ | non-metastatic |
| **H5** | metastasis​ | metastatic​ | metastasis​ | non-metastatic |
| **H6** | metastasis​ | metastasis​ | metastasis​ | non-metastatic |
| **H7** | metastasis​ | metastasis​ | non-metastatic | metastasis​ |
| **H8** | metastasis​ | metastasis​ | metastasis​ | non-metastatic​ |
| **L1** | metastasis​ | non-metastatic​ | metastasis​ | non-metastatic |
| **L2** | metastasis​ | non-metastatic​ | non-metastatic | non-metastatic |
| **L3** | non-metastatic​ | non-metastatic​ | metastasis​ ​ | non-metastatic ​ |
| **L4** | metastasis​ | non-metastatic​ | non-metastatic | non-metastatic |
| **L5** | metastasis​ | non-metastatic​ | metastasis​ | non-metastatic ​ |
| **L6** | non-metastatic​ | non-metastatic​ | non-metastatic | non-metastatic​ |

(H1–H8: AC093895.1 high-expression group; L1–L8: AC093895.1 low-expression group)
