# Supplementary material for: The long noncoding RNA AC093895.1 promotes ovarian cancer formation and metastasis through a positive feedback network dependent on the transcription factor SOX4
Source: Cell Death Dis. 2026 Feb 3;17(1):202. doi: 10.1038/s41419-026-08429-2 (PMC12894752; doi:10.1038/s41419-026-08429-2)
Supplement: Supplementary file 5 — original western blots [file 41419_2026_8429_MOESM5_ESM.pdf]

FIG1K A2780

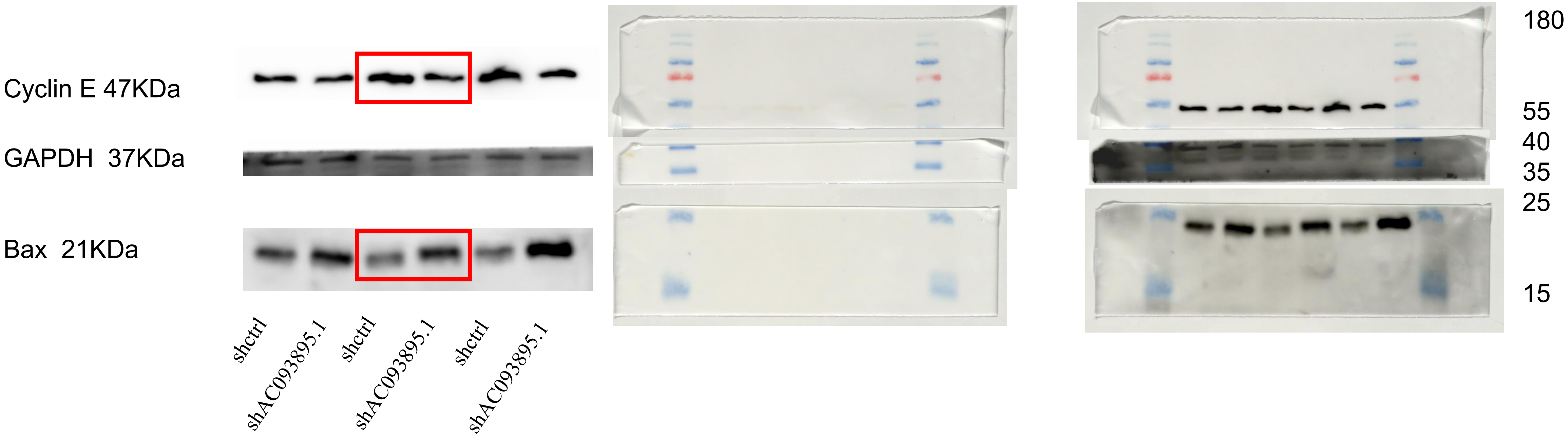

Cyclin E 47KDa
GAPDH 37KDa
Bax 21KDa
shctrl
shAC093895.1
shctrl
shAC093895.1
shctrl
shAC093895.1
180
55
40
35
25
15

FIG1K A2780

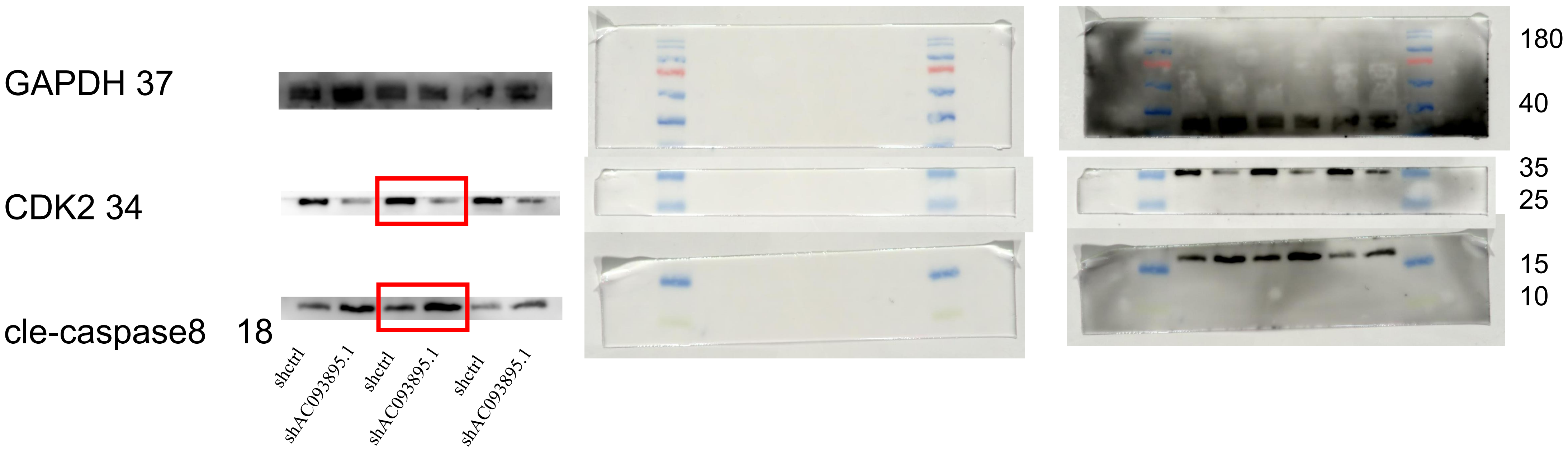

GAPDH 37
CDK2 34
cle-caspase8 18
shctrl
shAC093895.1
shctrl
shAC093895.1
shctrl
shAC093895.1
180
40
35
25
15
10

FIG1K A2780

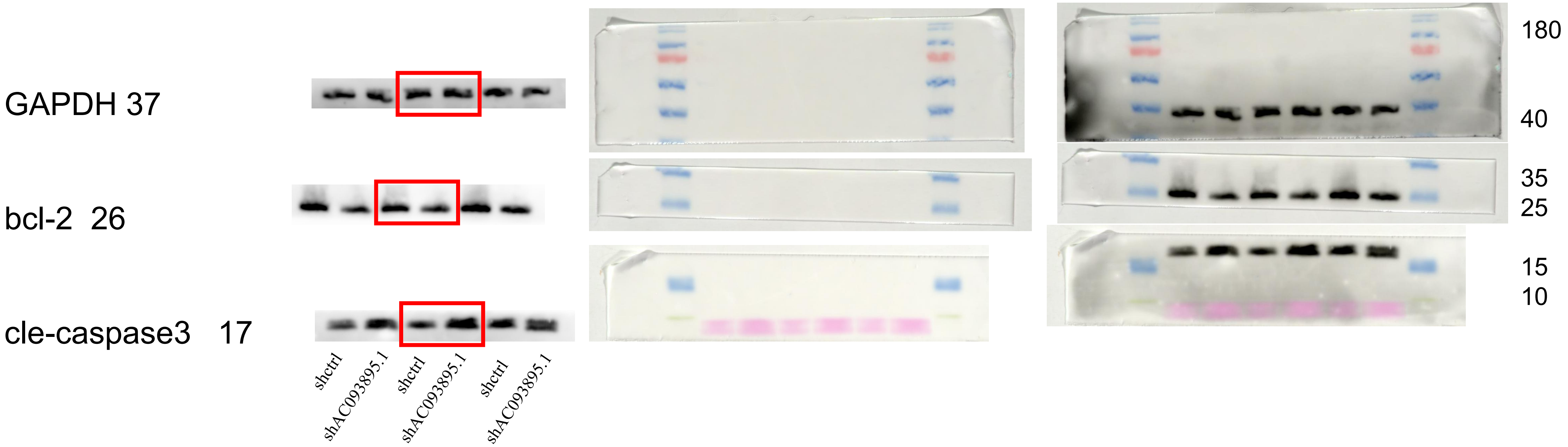
GAPDH 37
bcl-2 26
cle-caspase3 17
shctrl
shAC093895.1
shctrl
shAC093895.1
shctrl
shAC093895.1
180
40
35
25
15
10

FIG1K SKOV3

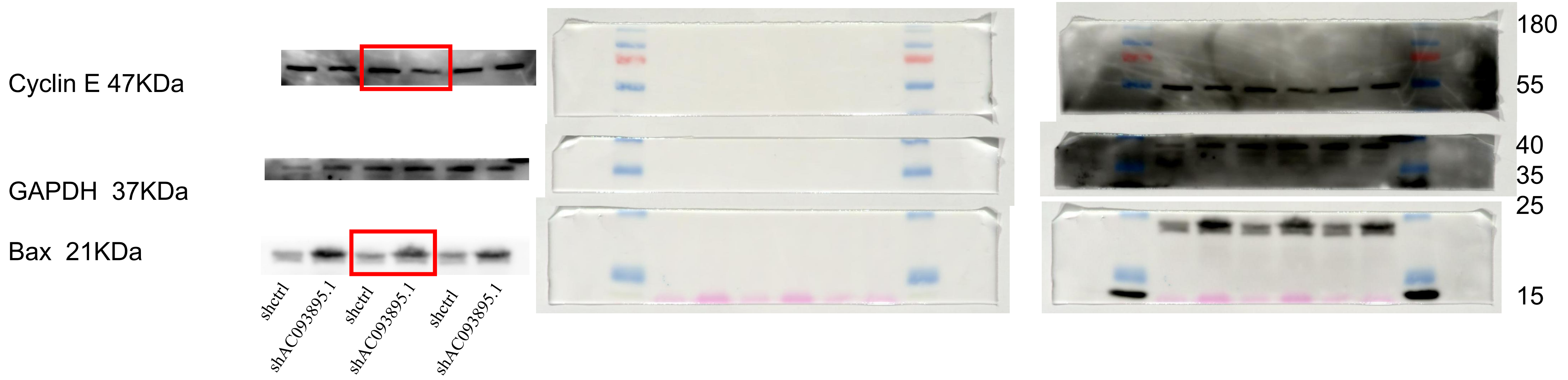
Cyclin E 47KDa
GAPDH 37KDa
Bax 21KDa
shctrl
shAC093895.1
shctrl
shAC093895.1
shctrl
shAC093895.1
180
55
40
35
25
15

FIG1K SKOV3

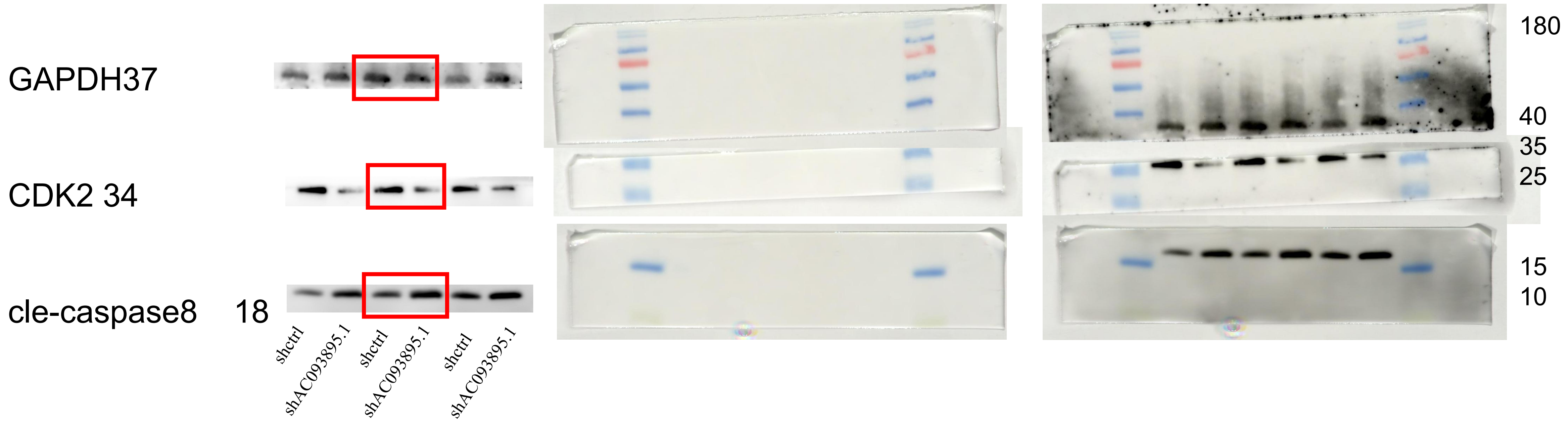

GAPDH37
CDK2 34
cle-caspase8
18
shctrl
shAC093895.1
shctrl
shAC093895.1
shctrl
shAC093895.1
180
40
35
25
15
10

FIG1K SKOV3

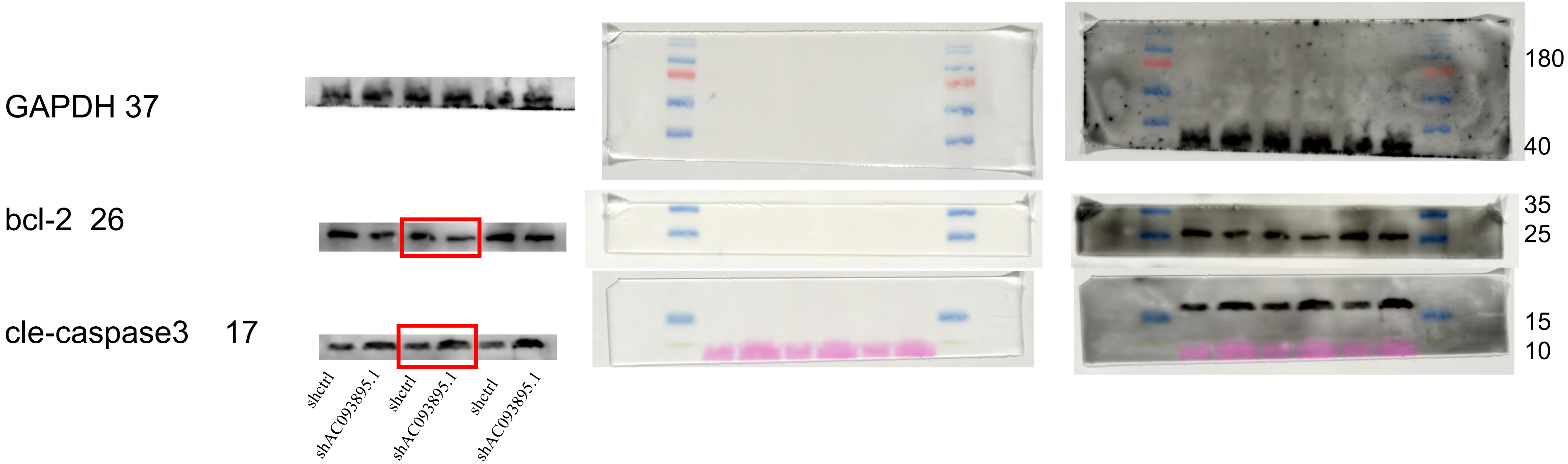

GAPDH 37
bcl-2 26
cle-caspase3 17
shctrl
shAC093895.1
shctrl
shAC093895.1
shctrl
shAC093895.1
180
40
35
25
15
10

FIG1P A2780

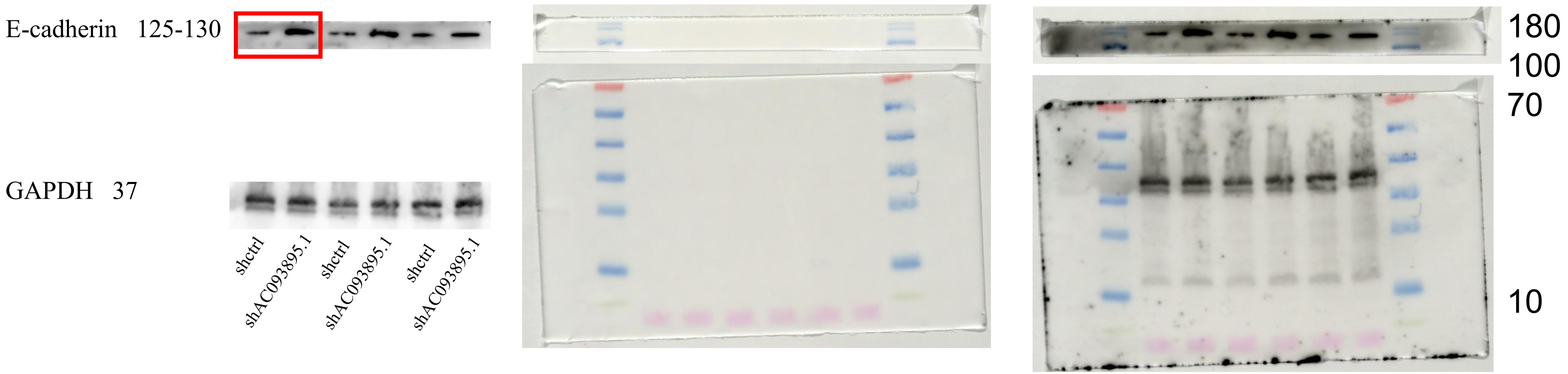

E-cadherin 125-130
GAPDH 37
shctrl
shAC093895.1
shctrl
shAC093895.1
shctrl
shAC093895.1
180
100
70
10

FIG1P A2780

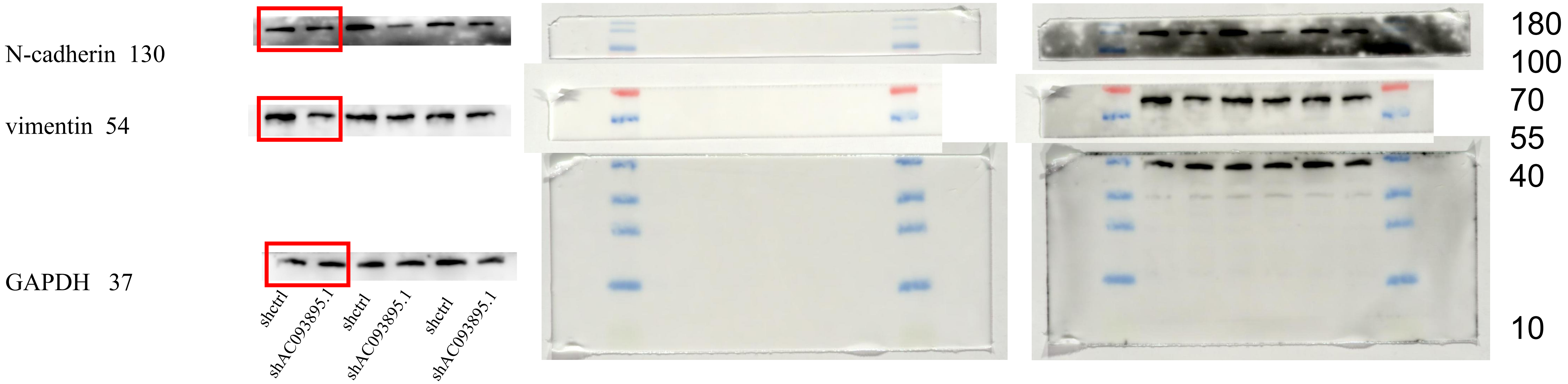
N-cadherin 130
vimentin 54
GAPDH 37
shctrl
shAC093895.1
shctrl
shAC093895.1
shctrl
shAC093895.1
180
100
70
55
40
10

FIG1P SKOV3

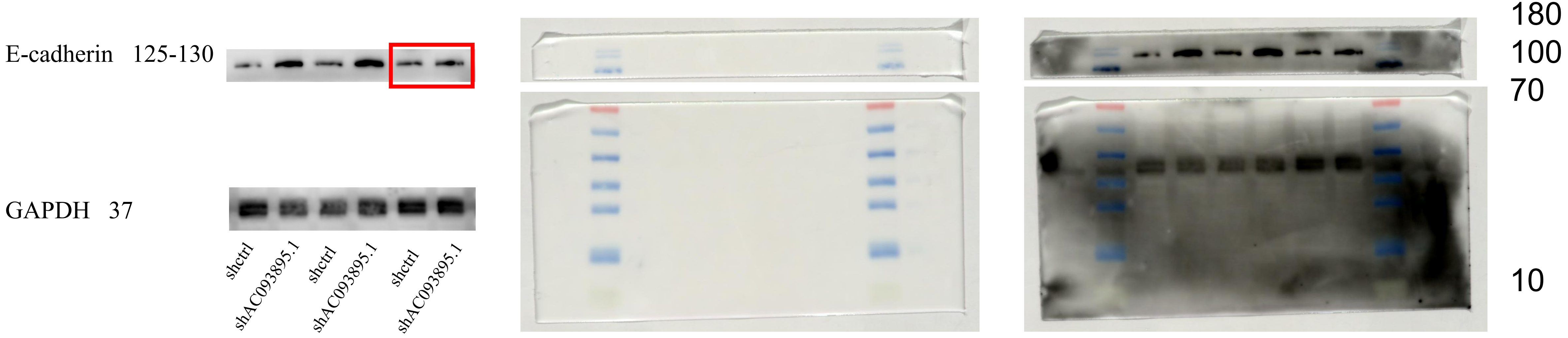

E-cadherin 125-130
GAPDH 37
shctrl
shAC093895.1
shctrl
shAC093895.1
shctrl
shAC093895.1
180
100
70
10

FIG1P SKOV3

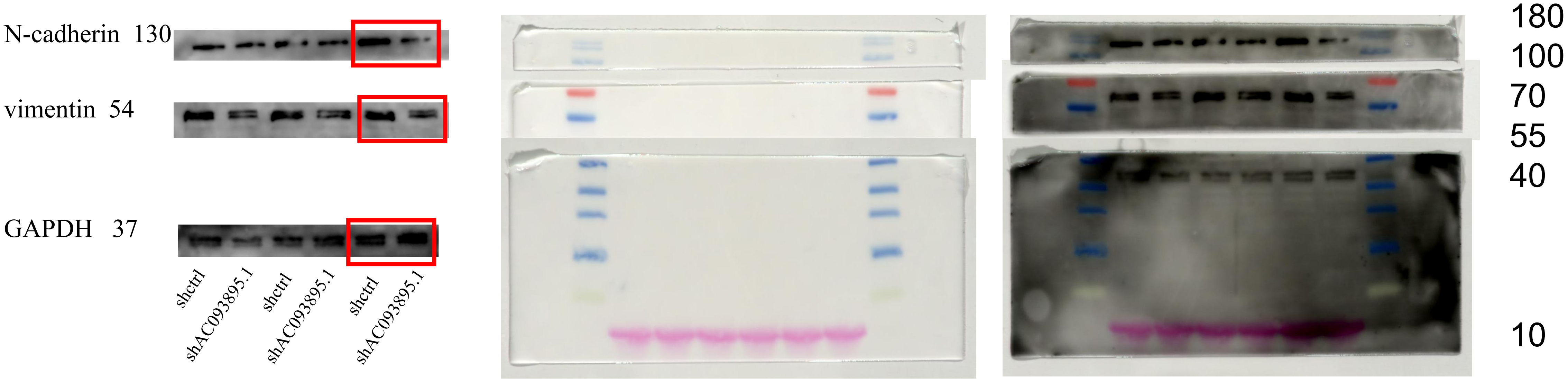
N-cadherin 130
vimentin 54
GAPDH 37
shctrl
shAC093895.1
shctrl
shAC093895.1
shctrl
shAC093895.1
180
100
70
55
40
10

FIG4I A2780

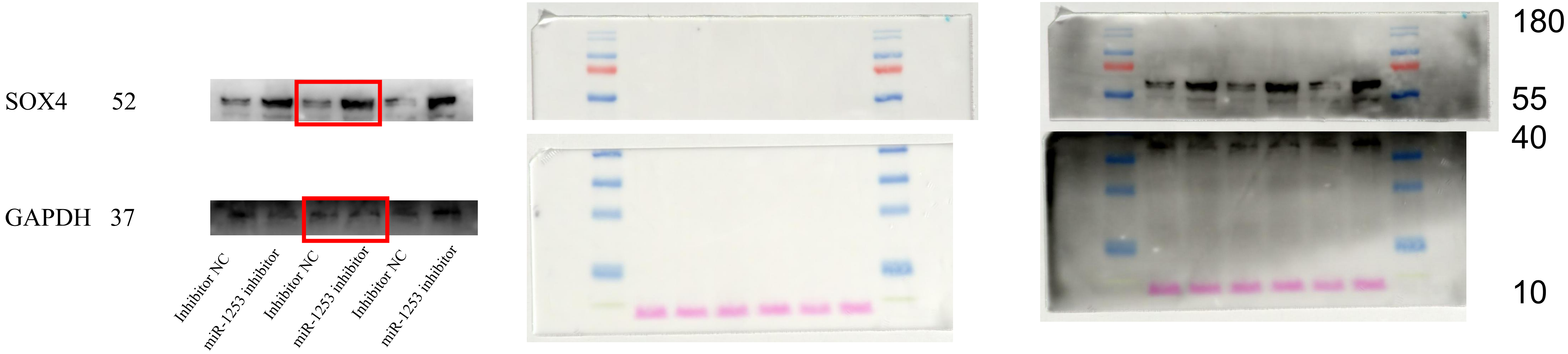

SOX4 52
GAPDH 37
Inhibitor NC
miR-1253 inhibitor
Inhibitor NC
miR-1253 inhibitor
Inhibitor NC
miR-1253 inhibitor
180
55
40
10

FIG4I SKOV3

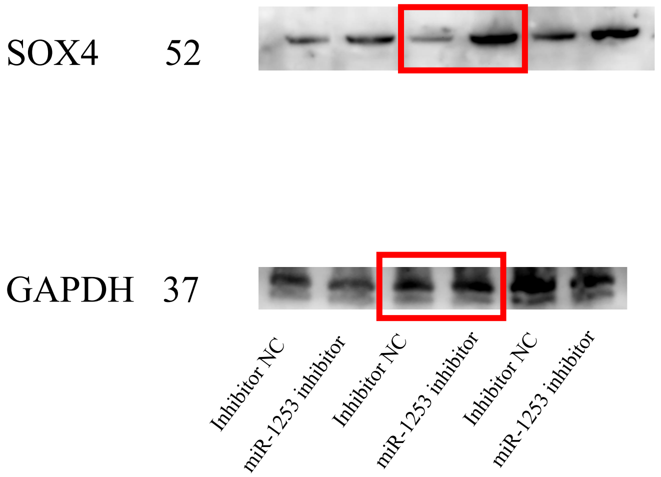
SOX4
52
GAPDH
37
Inhibitor NC
miR-1253 inhibitor
Inhibitor NC
miR-1253 inhibitor
Inhibitor NC
miR-1253 inhibitor

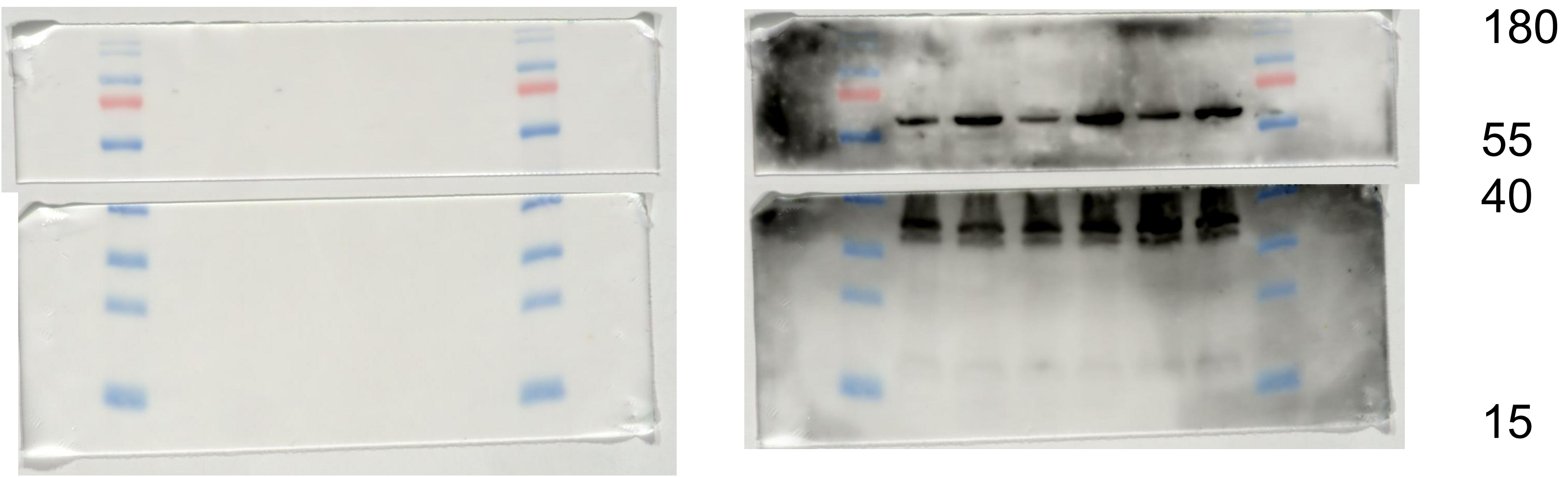
180
55
40
15

FIG5C A2780

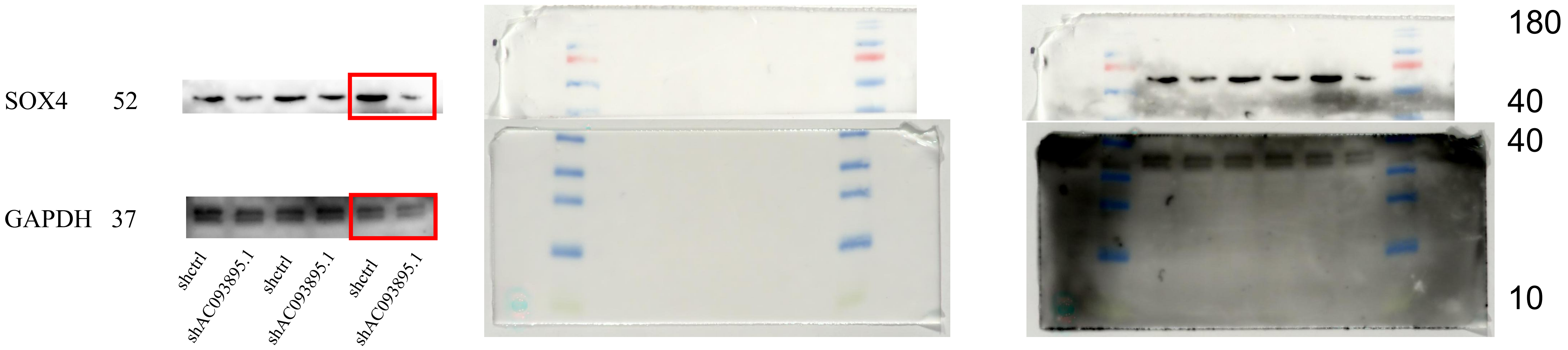
SOX4
52
GAPDH
37
shctrl
shAC093895.1
shctrl
shAC093895.1
shctrl
shAC093895.1
180
40
40
10

FIG5C SKOV3

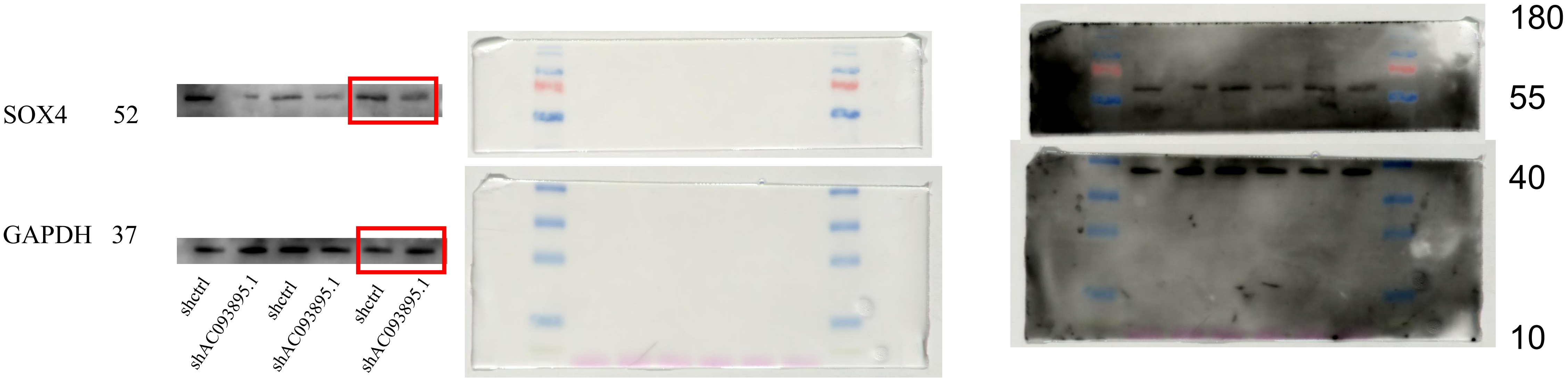
SOX4 52
GAPDH 37
shctrl
shAC093895.1
shctrl
shAC093895.1
shctrl
shAC093895.1
180
55
40
10

FIG6C A2780

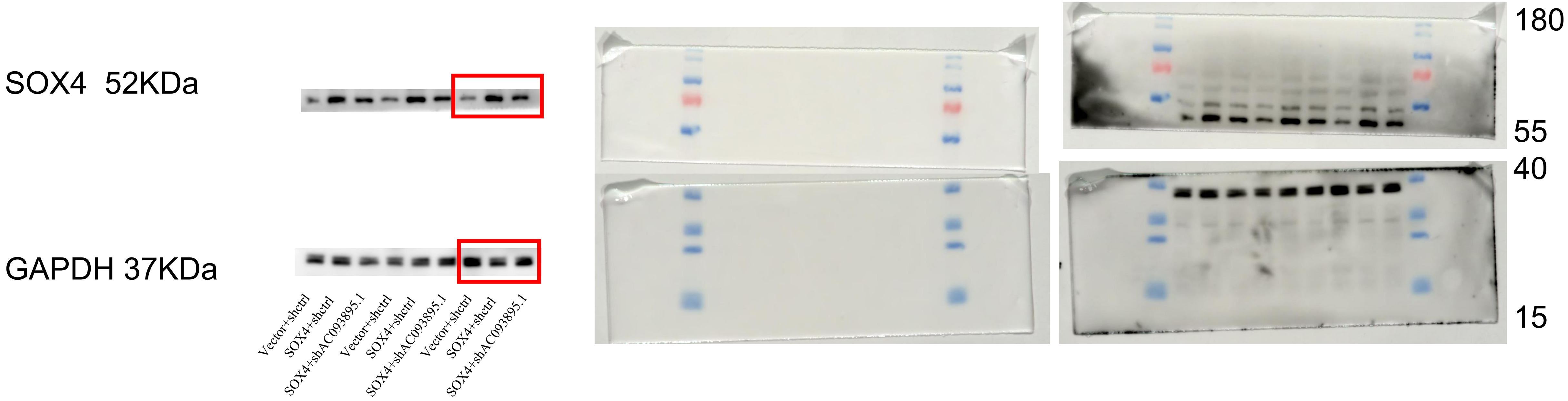

SOX4 52KDa
GAPDH 37KDa
Vector+shctrl
SOX4+shctrl
SOX4+shAC093895.1
Vector+shctrl
SOX4+shctrl
SOX4+shAC093895.1
Vector+shctrl
SOX4+shctrl
SOX4+shAC093895.1
180
55
40
15

FIG6C SKOV3

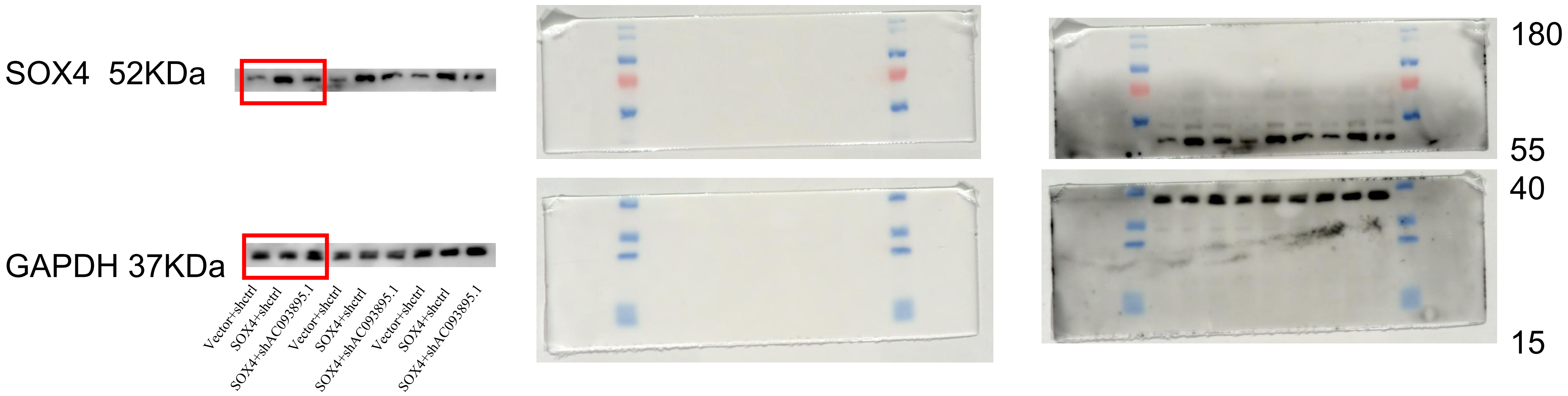
SOX4 52KDa
GAPDH 37KDa
Vector+shctrl
SOX4+shctrl
SOX4+shAC093895.1
Vector+shctrl
SOX4+shctrl
SOX4+shAC093895.1
Vector+shctrl
SOX4+shctrl
SOX4+shAC093895.1
180
55
40
15

fig8I

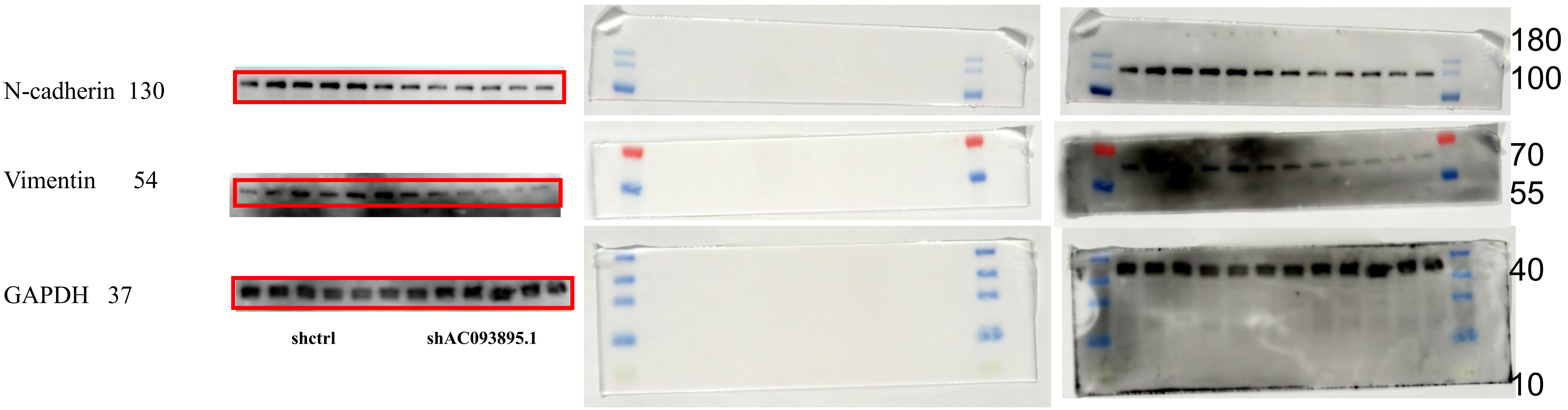

N-cadherin 130
Vimentin 54
GAPDH 37
shctrl
shAC093895.1
180
100
70
55
40
10

fig8I

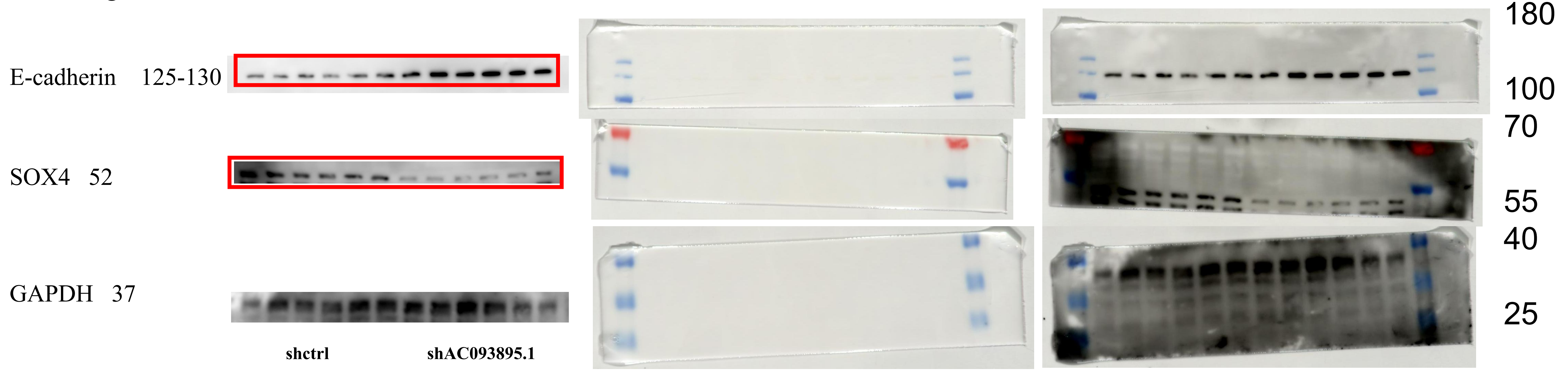
E-cadherin 125-130
SOX4 52
GAPDH 37
shctrl
shAC093895.1
180
100
70
55
40
25
